# De novo design of potent CRISPR–Cas13 inhibitors

**DOI:** 10.1038/s41589-025-02136-3

**Published:** 2026-01-26

**Authors:** Cyntia Taveneau, Her Xiang Chai, Jovita D’Silva, Rebecca S. Bamert, Honglin Chen, Brooke K. Hayes, Roland W. Calvert, Jacob Purcell, Daniel J. Curwen, Fabian Munder, Lisandra L. Martin, Jeremy J. Barr, Joseph Rosenbluh, Mohamed Fareh, Rhys Grinter, Gavin J. Knott

**Affiliations:** 1 Department of Biochemistry and Molecular Biology, Biomedicine Discovery Institute, Monash University, Clayton, Victoria Australia; 2 AI Protein Design Program, Biomedicine Discovery Institute, Monash University, Clayton, Victoria Australia; 3 Peter MacCallum Cancer Centre, Melbourne, Victoria Australia; 4 Sir Peter MacCallum Department of Oncology, The University of Melbourne, Parkville, Victoria Australia; 5 School of Chemistry, Monash University, Clayton, Victoria Australia; 6 Department of Microbiology, Biomedicine Discovery Institute, Monash University, Clayton, Victoria Australia; 7 School of Biological Sciences, Monash University, Clayton, Victoria Australia; 8 Functional Genomics Platform, Monash University, Melbourne, Victoria Australia; 9 Department of Biochemistry and Pharmacology, Bio21 Molecular Science and Biotechnology Institute, The University of Melbourne, Parkville, Victoria Australia; 10 Centre for Electron Microscopy of Membrane Proteins, Monash Institute of Pharmaceutical Sciences, Parkville, Victoria Australia

**Keywords:** Structural biology, Protein design, Microbiology

## Abstract

CRISPR–Cas systems are transformative tools for gene editing that can be tuned or controlled by anti-CRISPRs (Acrs)—phage-derived inhibitors that regulate CRISPR–Cas activity. However, Acrs that can inhibit biotechnologically relevant CRISPR systems are relatively rare and challenging to discover. To overcome this limitation, we describe a highly successful and rapid approach that leverages de novo protein design to develop new-to-nature proteins for controlling CRISPR–Cas activity. Here, using *Leptotrichia* *buccalis* CRISPR–Cas13a as a representative example, we demonstrate that Acrs designed using artificial intelligence (AIcrs) are capable of highly potent and specific inhibition of CRISPR–Cas13a nuclease activity. We present a comprehensive workflow for design validation and demonstrate AIcr functionality in controlling CRISPR–Cas13 activity in bacterial and human cells. The ability to design bespoke inhibitors of Cas effectors will contribute to the ongoing development of CRISPR–Cas tools in diverse applications across research, medicine, agriculture and microbiology.

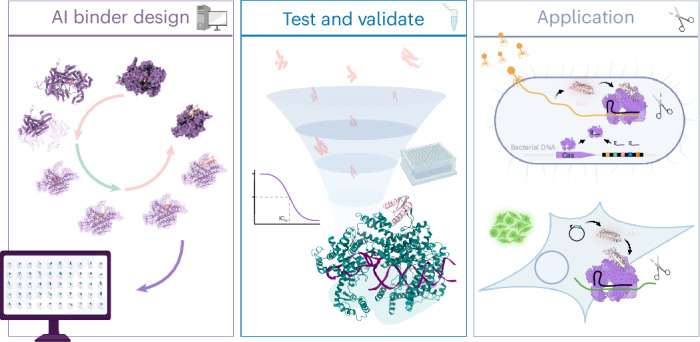

## Abstract (web summary)

Taveneau et al. leverage artificial-intelligence-driven protein design to create inhibitors that control RNA-targeting enzymes in cells, revealing a strategy to rapidly design off-switches for RNA-editing systems.

## Main

CRISPR–Cas effectors are microbial adaptive immune systems that carry out interference against mobile genetic elements^1^. CRISPR–Cas systems function through a Cas effector protein (typically nuclease) guided by a CRISPR RNA (crRNA) to base pair with foreign genetic material. Base pairing between the crRNA and the target nucleic acid triggers a conformational change in the Cas effector that activates the nuclease domain leading to target restriction^2^. CRISPR–Cas systems are categorized by their composition and broader mechanism of interference through a tiered classification of class (1 or 2), type (I, II, III, IV, V, VI or VII) and subtype^3,4^. Of particular interest are the class 2 CRISPR–Cas effectors (Cas9, Cas12 and Cas13)—enzymes that have transformed biotechnology with applications in precision gene editing, transcriptome engineering and diagnostics^5^. The widespread application of these systems underscores a need for effective tools to selectively control and tune their activity^6,7^.

**Fig. 1:**
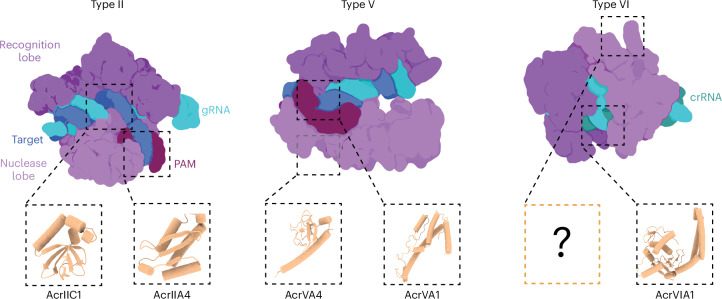
Phage-derived Acrs targeting type II CRISPR–Cas9, type V CRISPR–Cas12a and type VI CRISPR–Cas13a. Acr proteins use diverse mechanisms to inhibit CRISPR–Cas systems through interactions with the nuclease or recognition lobes. Targeting type II (Cas9), AcrIIA4 (PDB 5XN4)^57^ and AcrIIC1 (PDB 5VGB)^40^ block DNA target recognition and the HNH nuclease, respectively. Targeting type V (Cas12a), AcrVA4 (PDB 6NMA)^58^ allosterically inhibits DNA binding and AcrVA1 (PDB 6NMD)^58^ cleaves the crRNA to prevent target DNA recognition. Targeting type VI (Cas13), AcrVIA1 (PDB 6VRB)^14^ inhibits RNA cleavage by blocking aRNA recognition. No inhibitors have been described that block the highly conserved HEPN domain of type VI CRISPR–Cas13. PAM, protospacer-adjacent motif; gRNA, guide RNA; crRNA, CRISPR RNA; aRNA, activator RNA.

Anti-CRISPRs (Acrs) are mobile genetic element encoded proteins or RNAs that inhibit the activity of CRISPR–Cas machinery^8,9^. Originally discovered with the observation of resistance to CRISPR–Cas targeting^10^, studies have since leveraged guilt-by-association with known Acr-associated (*aca*) genes or evidence of self-targeting to uncover potent Acrs capable of disarming Cas9 (ref. ^11^), Cas12 (refs. ^12,13^) or Cas13 (refs. ^14–16^). To expand the toolbox of available inhibitors, researchers have turned to engineering, deep learning and structure-guided discovery to broaden target range or Acr potency^17–20^. Like the CRISPR–Cas systems they target, Acr nomenclature adheres to a tiered classification system of class and subtype (for example, AcrIIA4—the fourth inhibitor of type II-A CRISPR–Cas9). Many Acrs exert their inhibitory effects directly on the Cas effector nuclease by blocking crRNA or target nucleic acid binding or the conserved catalytic center of the nuclease (Fig. 1). Once bound, Acrs function through diverse mechanisms including the prevention or disruption of active effector complex formation^21^, crRNA degradation^22^ and competitive or allosteric inhibition^23,24^. A recently described example is AcrVIB1, which inhibits Cas13b by binding the apo enzyme and promoting the formation of nonproductive crRNA-bound complexes that are prone to RNase-mediated degradation^16^. The diverse toolbox of inhibitors supports the precision control of gene editing in human cells to limit unintended edits^6^, control gene drives in the environment^25,26^ or act as selectable markers in phage engineering^27^. However, the diversity of known CRISPR–Cas effectors far outstrips the number of functionally validated and potent Acrs. This is particularly evident for the type VI RNA-guided, RNA-targeting CRISPR–Cas13 systems, for which only three validated inhibitors have been described^14–17,28^, leaving many of the biotechnologically relevant Cas13 systems without Acrs.

To overcome limitations in the discovery of naturally occurring Acrs, we leveraged de novo protein design to create new-to-nature protein inhibitors of CRISPR–Cas, which we call artificial intelligence (AI)-designed Acrs (AIcrs). Using protein generation with RoseTTAFold-Diffusion (RFdiffusion)^29^ and inverse folding with ProteinMPNN^30^, we describe the design of AIcrs against *Leptotrichia* *buccalis* (Lbu) CRISPR–Cas13a—a representative CRISPR–Cas13 family member for which no known or validated Acrs exist. Through extensive validation, we show that AIcrs function through a mode of action consistent with their design and demonstrate potent inhibition of CRISPR activity in bacterial and human cells. AIcrs have the potential to overcome limitations of traditional discovery-based methods to provide effective and versatile inhibitors that enhance the safety, efficacy and control of gene editing.

## Results

### De novo design of Cas13 nuclease inhibitors

Phage-derived Acrs are highly structurally diverse and typically exploit core functions in CRISPR–Cas assembly, activation or catalytic activity. To explore the application of de novo protein design in generating potent Acrs, we targeted the well-studied and structurally characterized LbuCas13a for which no known natural Acrs exist^31^. Beyond its suitability as a proof of concept because of extensive structural and mechanistic characterization, LbuCas13a has been applied as a tool for programmable mRNA targeting and efficient phage genome editing^32^. Potent inhibitors of LbuCas13a would offer tools to temporally modulate nuclease function or reduce cytotoxicity in RNA knockdown applications.

**Fig. 2:**
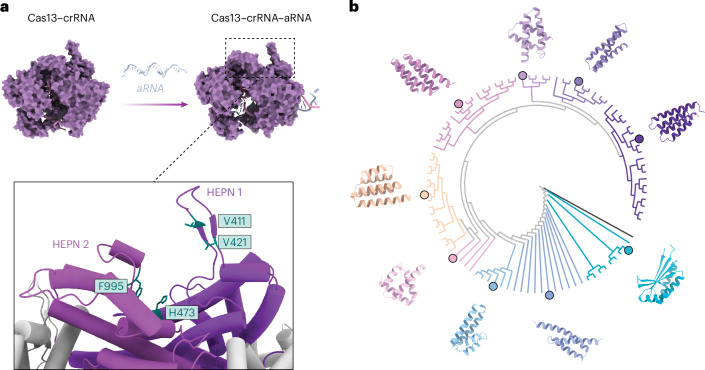
AIcr designs targeted to the LbuCas13a HEPN domain. **a**, Structure of LbuCas13a (surface, purple) bound to crRNA (pink) hybridized to an aRNA (pale blue) drives activation of the HEPN nuclease (boxed region) (PDB 5XWY and 5XWP)^31^. Design hotspots (stick representation, teal) are shown between HEPN1 (cartoon, dark purple) and HEPN2 domains (cartoon, light purple). **b**, MSTA tree for the 96 filtered AIcr designs rooted to GFP (black) and colored by structural class. Representative structurally diverse AIcrs are shown (cartoons at circled nodes).

**Extended Data Fig. 1:**
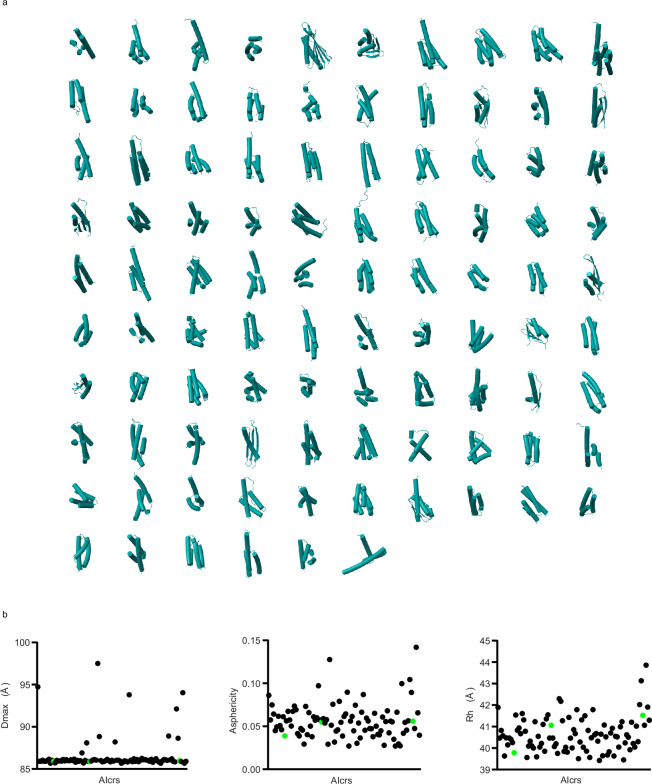
Designed AIcr fold diversity. **a**, AlphaFold2 predictions of the 96 AIcrs tested in this study (cartoon representations shown in teal). **b**, Dimensional and morphological diversity of the 96 AIcrs. Maximum diameter (Dmax, Å), asphericity, and hydration radius (Rh, Å) were calculated using the MDAnalysis Python package3. The majority of Dmax values are approximately 86 Å, asphericity ranges between 0.05 and 0.1, and Rh values are around 40 to 42 Å, indicating that the AI-designed structures exhibit consistent overall size and near-spherical morphology. The green dots refer to AIcrVIA1, AIcrVIA2 and AIcrVIA3.

To inhibit LbuCas13a activity, we generated designs against the higher eukaryotes and prokaryotes nucleotide-binding (HEPN) endoribonuclease domain, a domain that is strictly conserved across type VI CRISPR–Cas13 systems. The HEPN nuclease cleaves substrate RNA in response to crRNA hybridization to a complementary activator RNA (aRNA)^33,34^. Using RFdiffusion, we generated protein scaffolds to create AIcrs that would directly block substrate RNA recognition through interaction with the conserved HEPN nuclease active-site pocket at residue H473 and proximal surface-accessible hydrophobic residues (V411, V421 and F995) (Fig. 2a). From a set of 10,000 designs, we selected 96 candidates using a combination of in-house and previously published in silico metrics^29^. The final set of 96 AIcr designs were then analyzed by multiple-structure alignment (MSTA) (Fig. 2b and Supplementary Fig. 1). AIcr designs ranged from 70 to 127 aa in length with highly diverse structures, consistent with unconditional protein fold generation (Extended Data Fig. 1a). A search of the predicted AIcr structures against the Protein Data Bank (PDB)^35^ or AlphaFold Database^36,37^ using Foldseek^38^ revealed no significant similarity to known proteins. Examining the MSTA guide tree revealed several mixed αβ designs and numerous related clusters of predominantly α-helical designs (Fig. 2b and Extended Data Fig. 1a). Across the structurally distinct clusters and within structurally related clusters, there was no clear sequence conservation (Supplementary Fig. 1). All AIcrs filtered into the final set exhibited relatively globular shapes (Extended Data Fig. 1b) and were predicted to have negatively charged surface potentials (isoelectric points 3.6–4.9) (Supplementary Fig. 2), consistent with design against the positively charged HEPN nuclease and akin to many phage-derived Acrs. Examining AlphaFold2 predictions from each AIcr structural class in complex with the target Cas13 indicated diverse mechanisms of target hotspot engagement through charged or hydrophobic interactions, in addition to HEPN active-site shape complementarity (Supplementary Figs. 2–4). Collectively, these data support the potential application of AI protein design to generate highly compact Acr-like proteins directed to functionally relevant sites on CRISPR–Cas machinery.

### AIcrs inhibit Cas13 nuclease activity

**Fig. 3:**
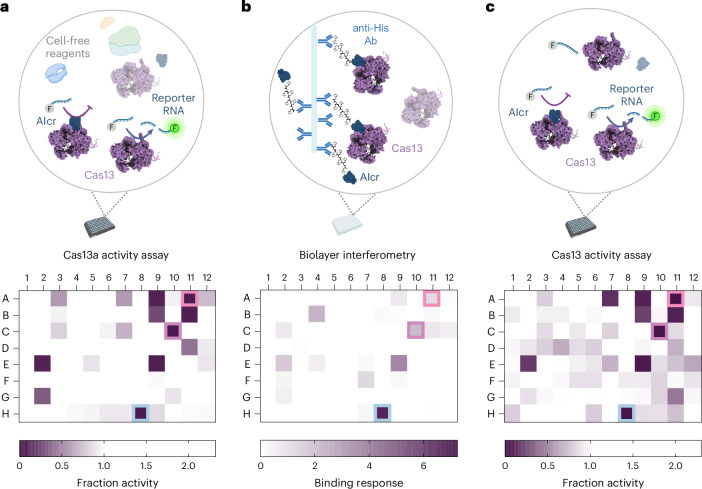
AIcrs are functional inhibitors of LbuCas13a activity. Schematic representations of the experiment (top row) and heat maps corresponding to a 96-well plate layout (bottom row), where each well contains a single designed AIcr. **a**, RNase activity of LbuCas13a–crRNA–aRNA in the presence of cell-free expressed AIcrs. Fluorescence values at 60 min are shown normalized to LbuCas13a–crRNA–aRNA activity alone. **b**, BLI binding assay measuring the interaction between LbuCas13a complexes and His–AIcrs, immobilized by an anti-His antibody. Maximum binding response height is shown normalized to the AIcr load response. **c**, RNase activity of LbuCas13a–crRNA–aRNA in the presence of purified AIcrs. Normalization and timing were as described in **a**. Three lead candidates (A11, C10 and H8) were selected for further characterization (boxed). Source data

**Extended Data Fig. 2:**
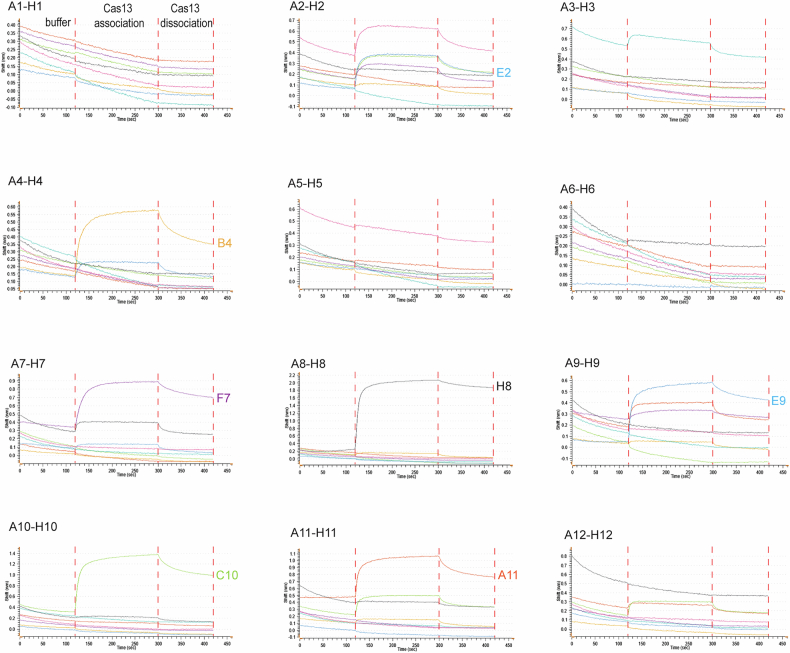
Raw biolayer interferometry data for AIcr binding to the LbuCas13a-crRNA-aRNA complex. Each experimental panel shows a plate column of eight AIcr designs (A1-H1, A2-H2, etc.) loaded onto Anti-His probes, followed by washing with buffer Q. The association phase is represented by binding of LbuCas13a-crRNA-aRNA, and the subsequent dissociation phase is shown after washing with buffer Q. The raw data were normalized by subtracting the signal from a ‘no AIcr bound’ control experiment using the same probes, and the difference is depicted for each phase (wash, association, and dissociation).

To rapidly screen each AIcr for the ability to inhibit LbuCas13a HEPN nuclease activity, we developed a cell-free expression and Cas13a activity assay system. Microgram quantities of unpurified cell-free expressed AIcrs were supplemented into an LbuCas13a HEPN nuclease activity assay containing LbuCas13a–crRNA complex, complementary aRNA and a fluorescence-quenched reporter substrate RNA (Fig. 3a). In the absence of AIcrs, activated LbuCas13a freely cleaved reporter RNA resulting in an increase in fluorescence (Supplementary Fig. 5). In the presence of cell-free expressed AIcrs, we observed a >50% reduction in fluorescence after 60 min for ten of the 96 designs, indicative of a reduction in LbuCas13a activity (Fig. 3a and Supplementary Fig. 5). To further investigate AIcr-mediated inhibition, we measured the association of LbuCas13a complexes (crRNA ± aRNA) to anti-His antibody immobilized AIcrs using biolayer interferometry (BLI) (Fig. 3b, Extended Data Fig. 2 and Supplementary Fig. 6). Broadly consistent with the coupled cell-free and Cas13a activity assay, seven AIcrs displayed binding to the activated LbuCas13a–crRNA–aRNA complex and, of these, five displayed binding to the LbuCas13a–crRNA complex (Extended Data Fig. 2 and Supplementary Fig. 6).

To validate our initial screen and account for any false negatives or positives in the cell-free (where AIcr abundance is not controlled) or BLI (where binder immobilization or orientation may introduce artifacts), we partially purified all 96 AIcr designs using batch-based affinity chromatography (Supplementary Fig. 7) and measured their ability to inhibit LbuCas13a HEPN nuclease activity at a known concentration. We observed near-perfect agreement between the inhibition activities of AIcrs generated in either the cell-free or semipurified setting (Fig. 3c and Supplementary Fig. 8), with differences likely stemming from assay sensitivity. Interestingly, from our set of 96 AIcrs, no single in silico parameter was predictive of success in vitro (Supplementary Fig. 9). With a success rate of 10% and an 8-week turnaround time from design to validated inhibition, the generation of AIcrs as inhibitors of CRISPR–Cas is a highly cost-effective, feasible and efficient alternative to the discovery of naturally encoded Acrs.

We selected three AIcrs (A11, C10 and H8) that displayed concordant binding (Fig. 3b) and enzyme inhibition activity (Fig. 3c) for further investigation and rigorous validation. Each AIcr was predicted to be structurally and mechanistically diverse in terms of their engagement with LbuCas13a HEPN (Supplementary Figs. 2–4). A11 adopts an elongated fold comprising both α-helices and β-sheets, enabling it to span the active-site cleft between the two HEPN domains. In contrast, C10 and H8 are predicted to adopt compact α-helical or β-stranded folds, respectively, that engage with HEPN2. In line with convention in the field and to differentiate them from naturally occurring phage-derived inhibitors, we named the three validated type VI LbuCas13a inhibitors AIcrVIA1 (A11), AIcrVIA2 (C10) and AIcrVIA3 (H8).

### AIcrVIAs are potent, stable and true to design

**Fig. 4:**
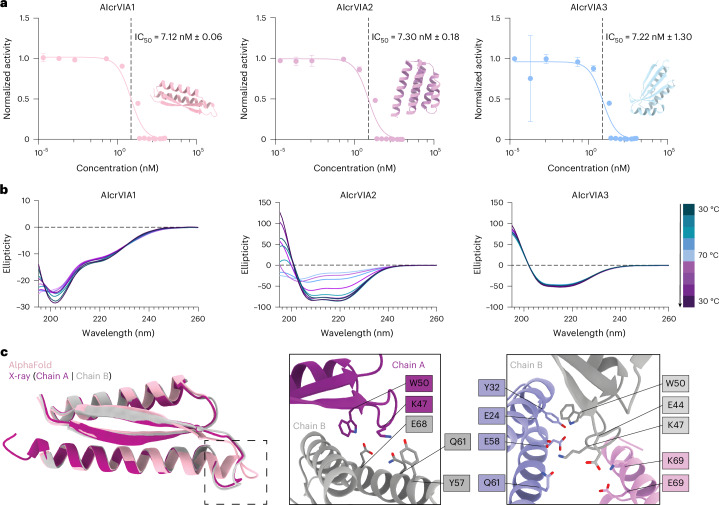
AIcrVIAs are potent inhibitors and true to design. **a**, Inhibitor dose dependence of LbuCas13a HEPN nuclease activity for AIcrVIA1 (left; pink), AIcrVIA2 (middle; dark pink) and AIcrVIA3 (right; blue). IC_50_ values (~7 nM) indicate high inhibitory potency. Data are shown as the mean ± s.d. (*n* = 3 technical triplicates). Cartoon representations for each AIcr design are shown. **b**, CD analysis of fold and stability of AIcrs across a temperature range. CD spectra of AIcrVIA1 (left), AIcrVIA2 (middle) and AIcrVIA3 (right) were recorded from 30 °C to 70 °C (blue gradient, heating) and back to 30 °C (purple gradient, cooling) in increments of 10 °C. The ellipticity curves reflect thermal stability and refolding behavior. **c**, X-ray crystal structure of AIcrVIA1 (cartoon, magenta and gray) superposed with the AlphaFold2 prediction (cartoon, pink), with the variable loop highlighted (dashed box). Right, the interaction interface for the variable loop is shown for each copy of within the asymmetric unit.

**Extended Data Fig. 3:**
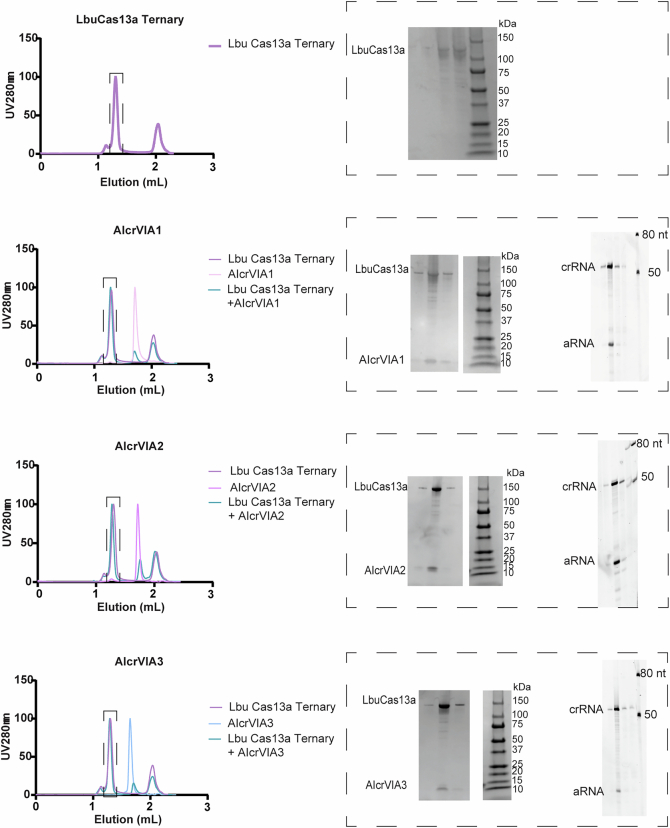
AIcrVIA1, AIcrVIA 2, and AIcrVIA3 interact with LbuCas13a. Gel filtration trace (left) and corresponding SDS-PAGE and urea gels (right) are shown for protein and RNA detection throughout the elution process. The black dashed rectangles highlight the fractions analyzed by SDS-PAGE and urea gel electrophoresis. Coelution of LbuCas13a with the AIcr binders indicates an interaction between the AIcrVIA designs (A1, A2, and A3) and LbuCas13a. Source data

To investigate the potency of AIcrVIA1–AIcrVIA3, we titrated each purified AIcrVIA against a fixed concentration of the LbuCas13a–crRNA–aRNA complex and measured the velocity of the activated HEPN nuclease (Fig. 4a). Calculating the concentration of AIcr required to achieve 50% inhibition (IC_50_) of LbuCas13a revealed values in the low nanomolar range for all three AIcrVIAs, consistent with high-affinity binding. To validate that the AIcrs reflected the overall structure of the designs, we measured the secondary-structure composition of each AIcrVIA using circular dichroism (CD) (Fig. 4b). CD spectra for the purified AIcrVIAs revealed secondary-structure fingerprints that reflect the designs with evidence for the mixed αβ and loop composition of AIcrVIA1, the exclusively α-helical composition of AIcrVIA2 and the predominantly β-stranded composition of AIcrVIA3. Moreover, consistent with other AI-designed proteins and the naturally occurring Acrs, AIcrVIAs were highly robust and thermostable (Fig. 4b). Having purified all AIcrVIAs to homogeneity using size-exclusion chromatography (SEC), we observed that each AIcrVIA eluted at a volume broadly consistent with the designed monomer (Extended Data Fig. 3).

To further validate congruence between design and reality, we successfully determined the X-ray crystal structure of AIcrVIA1 to 1.9-Å resolution (Fig. 4c and Supplementary Table 1). AIcrVIA1 crystallized with two copies in the asymmetric unit, each sharing a βαβα topology and an overall root-mean-square deviation (r.m.s.d.) of 0.64 Å (chain A to B). Consistent with the AlphaFold2 prediction, both copies of AIcrVIA1 resembled the designed structure (r.m.s.d. of 1.7 Å and 1.8 Å, His tag excluded for chain A and B, respectively). The only deviations between the AlphaFold2 model and the experimental structure resulted from extensive crystal contacts in residues 47–52 (Fig. 4c). Taken together, these biophysical and structural data provide clear evidence that AIcrVIAs are highly potent and stable and their structures reflect their intended design.

### AIcrVIAs are specific LbuCas13a HEPN nuclease inhibitors

**Fig. 5:**
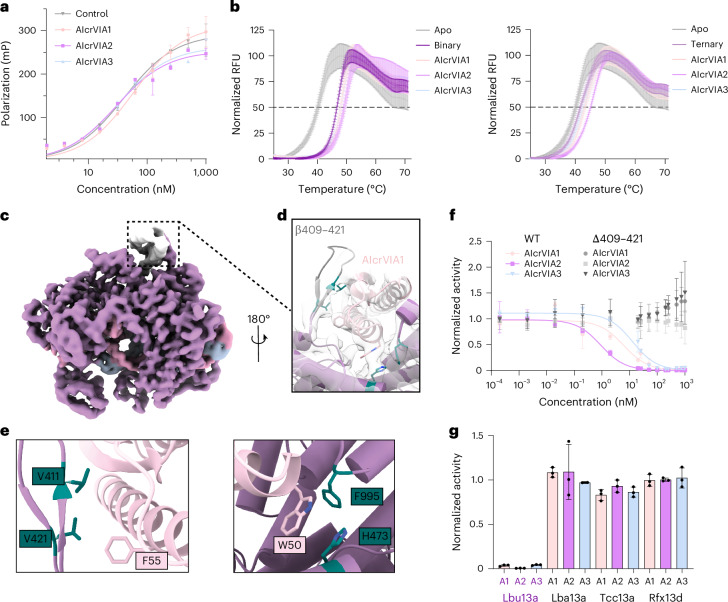
AIcrVIAs are competitive inhibitors of the LbuCas13a HEPN nuclease. **a**, Fluorescence polarization assessing aRNA binding to LbuCas13a–crRNA in the presence or absence (control) of AIcrVIAs (mean ± s.d., *n* = 3 technical triplicates). **b**, Melting curves of LbuCas13a–crRNA (left) and LbuCas13a–crRNA–aRNA (right) with and without AIcrVIs. Apo, LbuCas13a; binary, LbuCas13a–crRNA; ternary, LbuCas13a–crRNA–aRNA (mean ± s.d., *n* = 3 technical replicates). **c**, Cryo-EM reconstruction of the LbuCas13a ternary complex (crRNA in pink, aRNA in blue and LbuCas13a in purple) with AIcrVIA1 (gray). **d**, Cryo-EM structure of AIcrVIA1 (pink) interacting with the HEPN (purple) hotspot residues (teal sticks) and the β-turn loop (residues 409–421, black). **e**, LbuCas13a hotspots (sticks, teal) interacting with AIcrVIA1 aromatic residues (F55 and W50). **f**, LbuCas13a HEPN nuclease activity normalized to no inhibitor, comparing wild-type or LbuΔ409–421 in the presence of AIcrVIAs (mean ± s.d., *n* = 3 technical triplicates). **g**, Activity of diverse Cas13 incubated with AIcrVIA (mean ± s.d., *n* = 3 technical triplicates). Lbu13a, LbuCas13a; Lba13a, LbaCas13a; Tcc13a, TccCas13a; Rfx13d, RfxCas13d; A1, AcrVIA1; A2, AIcrVIA2; A3, AIcrVIA3 (mean ± s.d., *n* = 3 technical triplicates).

AIcrVIA1–AIcrVIA3 were designed to competitively inhibit the LbuCas13a nuclease through direct association with the conserved HEPN. To demonstrate this mode of inhibition, we first assayed aRNA binding to the LbuCas13a–crRNA complex using fluorescence anisotropy in the presence or absence of an excess of each AIcr (Fig. 5a). With an excess of AIcrVIA in competition with the crRNA and aRNA, binding to a complementary aRNA was unperturbed suggesting the absence of any nonspecific associations that would compete with crRNA or aRNA association. We next validated that each AIcrVIA was capable of a direct interaction with the activated LbuCas13a–crRNA–aRNA complex by SEC and thermal melt analysis. Consistent with our BLI binding data, each AIcrVIA stably eluted with the activated LbuCas13a complex (Extended Data Fig. 3) and the presence of any AIcrVIA stabilized both the LbuCas13a–crRNA or LbuCas13a–crRNA–aRNA complex (Fig. 5b).

**Extended Data Fig. 4:**
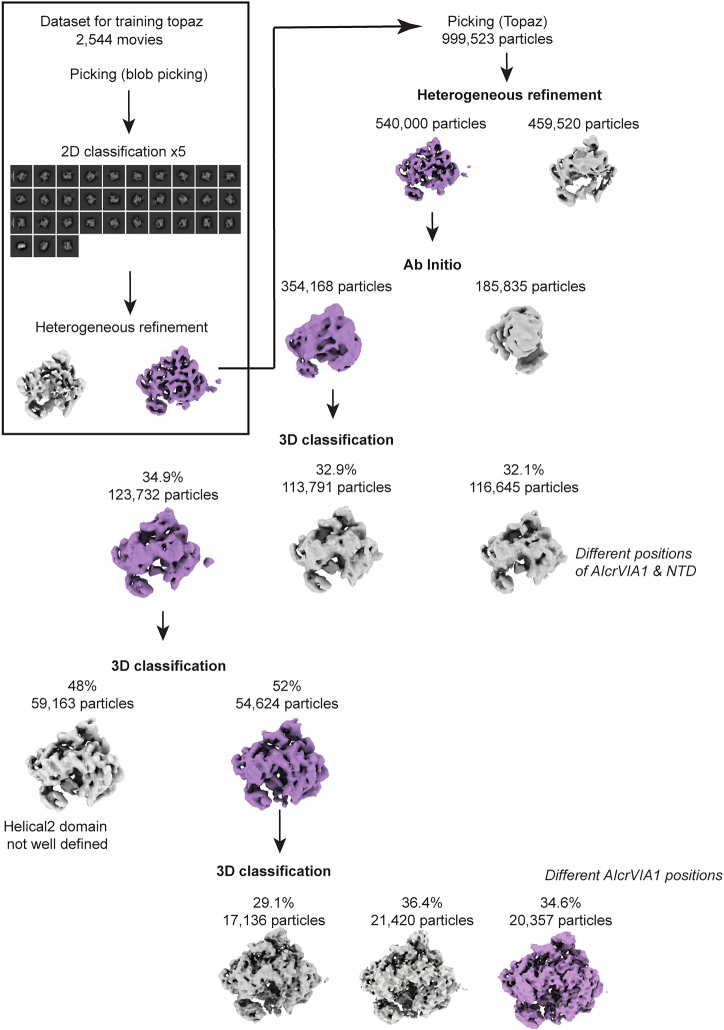
Step-by-Step Cryo-EM Workflow for data processing. A detailed schematic of the cryo-electron microscopy (cryo-EM) data processing pipeline for LbuCas13a-crRNA-aRNA in complex with AIcrVIA1.

To visualize an AIcrVIA in the act of inhibiting LbuCas13a, we determined a 3.55-Å cryo-electron microscopy (cryo-EM) structure of the activated wild-type LbuCas13a complex in the presence of AIcrVIA1 (Fig. 5c, Extended Data Table 1, Extended Data Fig. 4 and Supplementary Figs. 10 and 11). The overall structure of the activated LbuCas13a within the complex resembled that of the previously published X-ray crystal structure with an overall r.m.s.d. of 1.4 Å (PDB 5XWP; Supplementary Fig. 11a). We observed clearly resolved secondary-structure features for AIcrVIA1 within the HEPN nuclease, albeit with limited high-resolution information because of local conformational heterogeneity driven by continuous shifts in AIcrVIA1 around the HEPN nuclease (Supplementary Video 1). Despite the apparent dynamics, the resolved cryo-EM structure closely matched the final prediction for the complex LbuCas13a–AIcrVIA1 obtained from the AI design workflow, with an overall r.m.s.d. of 1.1 Å (Supplementary Fig. 11b,c). AIcrVIA1 is positioned within the HEPN nuclease, making numerous contacts with structural elements proximal to the conserved active site, including a highly positively charged β-stranded element (residues 409–421) (Fig. 5d and Supplementary Fig. 12a,b). Closer examination of the interaction interface revealed regions of AIcrVIA1 near the designated HEPN nuclease hotspots, illustrative of a congruence between design and experimental reality (Fig. 5e and Supplementary Fig. 12c).

Given the observed extensive contacts between AIcrVIA1 and the LbuCas13a β-stranded element (residues 409–421), we generated a deletion construct (LbuΔ409–421) to validate the observed interactions. As previously reported^31^, deletion of this β-stranded element (residues 409–421) reduces but does not abolish the collateral activity of LbuCas13a, effectively allowing us to assess the functional impact of the deletion on AIcr efficacy. Assaying LbuΔ409–421 HEPN nuclease activity in the presence of AIcrVIA1 revealed a complete loss of inhibition efficacy, supporting the observed interactions (Fig. 5f). Moreover, the inhibition activities of AIcrVIA2 and AIcrVIA3 were completely abolished against LbuΔ409–421, suggesting that these AIcrs likely occupy the same HEPN nuclease pocket, consistent with their design against the same features and hotspots (Fig. 5f). In line with these data, both AIcrVIA1 and AIcrVIA2 could not be copurified with activated LbuΔ409–421, whereas AIcrVIA3 maintained some limited association, collectively suggesting the loss of a stable interaction with the deletion of the β-stranded element (Supplementary Fig. 13). Taken together, these findings point to a highly specific mechanism of substrate RNA-competitive inhibition at the HEPN nuclease for all three AIcrVIAs.

**Extended Data Fig. 5:**
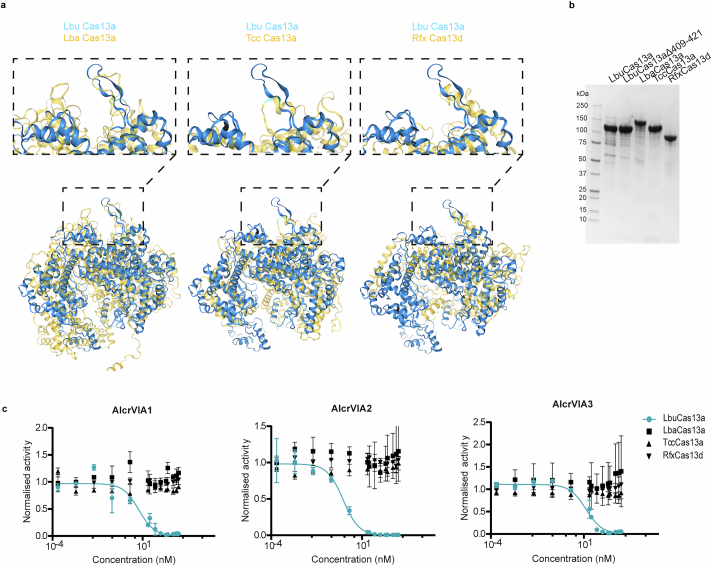
AIcrVIA1, AIcrVIA2, and AIcrVIA3 are specific to LbuCas13a. **a**, Superposition of LbuCas13a with LbaCas13a, TccCas13a, and RfxCas13d structures using FoldMason^60^. **b**, LbuCas13a, LbaCas13a, TccCas13a, and RfxCas13d proteins purified for this study with the purity of all Cas13 proteins confirmed by SDS-PAGE analysis. **c**, Activity assays were conducted for LbuCas13a, LbaCas13a, TccCas13a, and RfxCas13d in the presence of AIcrVIA1 (left), AIcrVIA2 (right), or AIcrVIA3 (left). While the nuclease activity of LbuCas13a is nearly abolished by these AIcrVIA inhibitors, the activity of the other Cas13 proteins remains unaffected, demonstrating the specificity of inhibition. Data are shown as mean ± s.d. (n = 3, technical triplicates). Source data

Target selectivity is critical to the application of Acrs. Most phage-derived Acrs are highly specific to their target Cas effector, with only a few examples of broad-spectrum Acrs^22,39,40^. To investigate target selectivity and validate AIcrVIAs as narrow-spectrum HEPN nuclease inhibitors, we first compared LbuCas13a to a set of diverse but functionally related enzymes: *Lachnospiraceae* *bacterium* (Lba*)* Cas13a, *Thermoclostridia* *caenicola* (Tcc) Cas13a and *Ruminococcus* *flavefaciens* (Rfx) Cas13d. Superposition of LbuCas13a with LbaCas13a, TccCas13a and RfxCas13d revealed near identical coordination of the active-site residues but substantial differences in structural elaborations proximal to the HEPN nuclease core that would likely prevent inhibitor binding (Extended Data Fig. 5a). To validate this and demonstrate specificity, we tested AIcrVIA activity across a range of concentrations in excess of the active Cas13 enzyme, which revealed no apparent inhibition of LbaCas13a, TccCas13a or RfxCas13d, indicative of high design specificity (Fig. 5g and Extended Data Fig. 5b,c). Combined with their validated potency, the observed specificity underscores the designed selectivity of AIcrVIAs and supports their potential for targeted inhibition in biotechnological applications.

### AIcrs inhibit Cas13a-mediated activity in cells

**Fig. 6:**
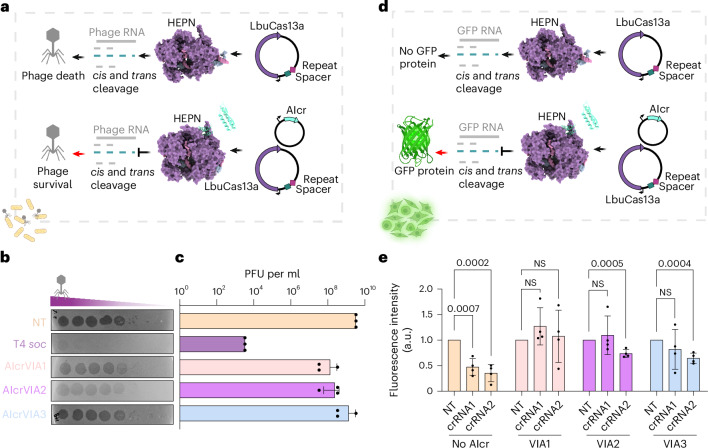
AIcrs inhibit Cas13 in bacterial and human cells. **a**, Phage assay to assess AIcrVIA efficacy in *E*. *coli* expressing LbuCas13a–crRNA. Induction of LbuCas13a enables defense against T4 phages, whereas induction of AIcr inhibits LbuCas13a activity to allow T4 replication. **b**, Phage plaque assay performed on *E*. *coli* coexpressing LbuCas13a and crRNA targeting T4 *soc*, incubated with serial dilutions of T4 phage (decreasing concentration from left to right). **c**, Quantified EOP, represented as PFU per ml, for each AIcrVIA (mean ± s.d., *n* = 3 biological replicates). **d**, Human cell fluorescence assay used to evaluate AIcrVIA efficacy. HEK293T cells were cotransfected with plasmids encoding LbuCas13a, GFP-targeting or nontargeting crRNA and AIcrVIA constructs. **e**, Quantification of GFP fluorescence normalized to nontargeting (NT) in the presence of GFP-targeting crRNAs (crRNA1 and crRNA2) (mean ± s.d., *n* = 4 biological replicates). Statistical significance was determined using unpaired *t*-tests; *P* values are indicated on the graph. NS, not significant.

**Extended Data Fig. 6:**
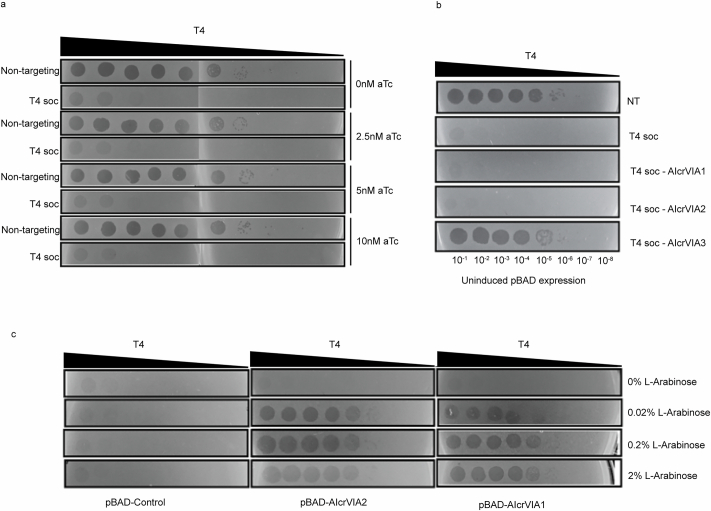
Optimization of protein expression conditions for the phage assay. **a**. Plaque assay optimization of LbuCas13a expression and crRNA production in E. coli using aTc induction. This assay was performed with a plasmid carrying an aTc-inducible LbuCas13a vector and either a constitutively expressed non-targeting (NT) crRNA or a T4 soc-targeting crRNA. No toxicity was observed upon induction. (***NT***: *non-targeting crRNA*; ***T4 soc***: *T4 soc-targeting crRNA*.) **b**, LbuCas13a inhibition by AIcrs under uninduced conditions using plaque assay. **c**. Optimization of AIcrVIA1 and AIcrVIA2 expression with increasing arabinose concentrations. Results indicated that 2% arabinose provided optimal expression levels. Optimization of AIcrVIA3 was not performed, as 2% arabinose was deemed sufficient based on these results.

**Extended Data Fig. 7:**
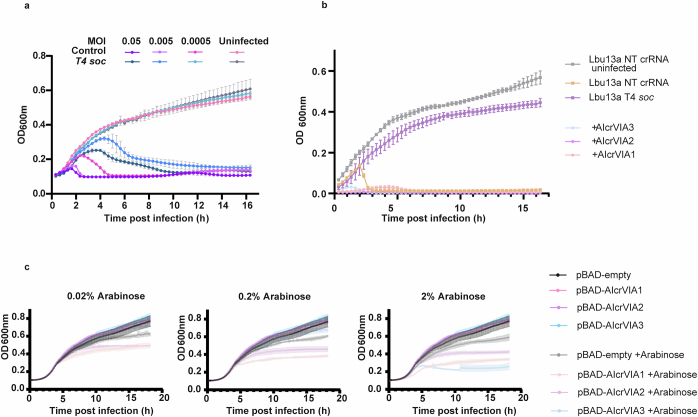
Phage liquid culture assay with LbuCas13a and AIcrVIAs. **a**, Determination of the multiplicity of infection (MOI) required for LbuCas13a to confer resistance against T4 phage infection in liquid culture. The optimal MOI was identified by monitoring infection dynamics and escape efficiency. **b**, Phage liquid culture assay demonstrating reduction in E. coli growth in the presence of AIcrVIAs, confirming inhibition of LbuCas13a. Data from b are shown as mean ± s.d. (n = 3, independent experiments). Data are shown as mean ± s.d. (n = 3 independent experiments). NT = non-targeting crRNA; T4 soc = T4 soc-targeting crRNA; PFU = plaque-forming units. **c**, Growth curves of E. coli strains carrying either empty vector or arabinose-inducible plasmids expressing AIcrVIA1, AIcrVIA2, or AIcrVIA3 across a range of arabinose concentrations (0.02%, 0.2%, and 2%). AIcrVIA1 and AIcrVIA2 displayed similar growth profiles across all concentrations, indicative of well-tolerated expression. AIcrVIA3 showed a clear growth defect at 2% arabinose, suggesting toxicity at high expression levels.

Phage-derived Acrs are highly successful tools to control the activity of CRISPR–Cas systems in bacterial and human cells^6,7^. To explore the applicability of AIcrVIAs in bacterial cells, we first constructed an anhydrous tetracycline (aTc)-inducible LbuCas13a vector with a constitutively expressed nontargeting or T4 *soc-*targeting crRNA as previously described (Fig. 6a)^32^. *Escherichia* *coli* expressing LbuCas13a and T4 *soc-*targeting crRNA effectively restricted T4 phage replication, whereas nontargeting crRNA had no effect (Fig. 6b,c), consistent with the RNA-guided HEPN nuclease activity of CRISPR–Cas13. Next, we constructed an arabinose-inducible AIcrVIA expression vector, which was cotransformed with the LbuCas13a–crRNA targeting or nontargeting vector (Fig. 6a). In contrast to the conditions lacking AIcr, the inducible expression of either AIcrVIA1, AIcrVIA2 or AIcrVIA3 recovered T4 phage titers across a range of induction strengths, demonstrating potent inhibition of LbuCas13a activity in *E*. *coli* (Fig. 6b,c and Extended Data Figs. 6 and 7). Examining the effect of AIcrVIAs in phage liquid culture assays using the same inducible system revealed a drastic reduction in *E*. *coli* growth in the presence of T4 phage and AIcrVIAs (Extended Data Fig. 7). These data demonstrate that the AIcrVIAs fulfill the same biological function as natural phage-derived Acrs and provide a strong indication for their applied potential in phage engineering.

**Extended Data Fig. 8:**
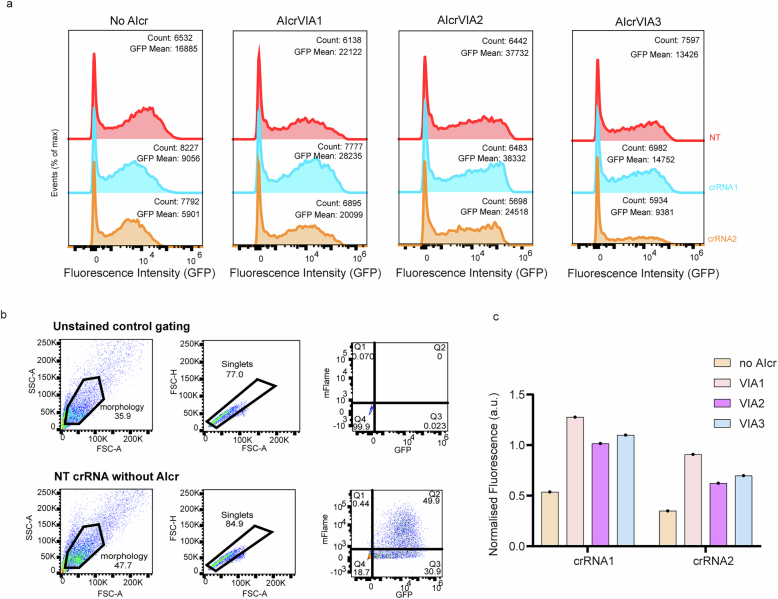
Flow cytometry analysis of GFP expression in HEK293T cells for AIcrVIA functional validation. **a**, Flow cytometry histograms showing GFP fluorescence in HEK293T cells co-transfected with LbuCas13a, GFP-targeting crRNAs (crRNA1 or crRNA2), or a non-targeting crRNA (NT), with or without AIcrVIAs (VIA1–3). **b**, Flow Cytometry gating strategy. **C**, Quantification of GFP fluorescence normalised to the NT control within each condition. Fluorescence suppression by GFP-targeting crRNAs is rescued upon co-expression of AIcrVIAs, consistent with inhibition of LbuCas13a activity.

**Extended Data Fig. 9:**
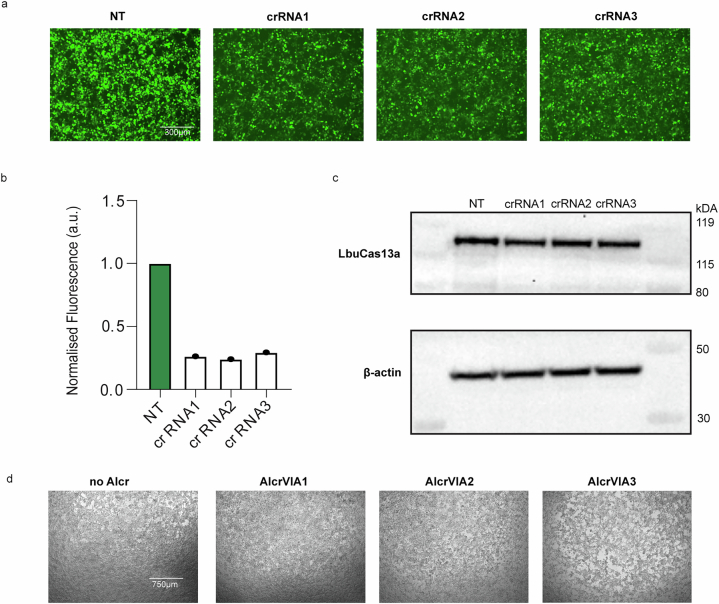
Validation and optimization of the HEK293 fluorescence assay. **a**, Representative fluorescence microscopy images of HEK293T cells co-transfected with lentiviral constructs expressing LbuCas13a and either non-targeting crRNA (NT) or targeting crRNAs (crRNA1, crRNA2, crRNA3) against GFP. **b**, Quantification of GFP fluorescence from image analysis reveals a robust decrease in GFP signal upon expression of targeting crRNAs, confirming effective Cas13-mediated RNA silencing. crRNA1 and crRNA2 were selected for downstream AIcrVIA experiments based on performance. **c**, Western blot analysis of LbuCas13a and β-actin from transfected cells confirms comparable Cas13a expression levels across all crRNA conditions. **d**, Representative images of HEK293T cells expressing AIcrVIA1, AIcrVIA2, or AIcrVIA3. While AIcrVIA1 and AIcrVIA2 show confluence comparable to controls, AIcrVIA3 expression results in an evident cytotoxic phenotype (n = 4, biological replicates). Source data

We next examined whether AIcrVIAs can modulate LbuCas13a activity in human cells, where differences in cellular context and toxicity potential pose distinct challenges^41^. To evaluate the functionality of AIcrVIAs in human cells, we established a fluorescence-based RNA silencing assay in HEK293T cells expressing LbuCas13a. Cells were cotransfected with LbuCas13a, GFP and mCherry reporter and a plasmid encoding either a nontargeting or GFP-targeting crRNA (crRNA1 and crRNA2) (Fig. 6d). In the presence of targeting crRNAs, we observed a robust ~50% reduction in fluorescence signal relative to nontargeting controls, consistent with Cas13a-mediated RNA cleavage of GFP mRNA with no observable collateral activity on mCherry mRNA (Fig. 6e, Extended Data Fig. 8 and Supplementary Fig. 14). To test whether AIcrVIAs can inhibit Cas13a activity in this context, HEK293T cells were cotransfected with AIcrVIA1, AIcrVIA2 or AIcrVIA3 expression plasmids, together with LbuCas13a, GFP reporter and crRNAs (Fig. 6d). Quantification of fluorescence intensity 48 h after transfection revealed that all three AIcrVIAs significantly restored GFP expression compared to targeting-only conditions (Fig. 6e), demonstrating effective inhibition of Cas13a in human cells. These results were further supported by fluorescence-activated cell sorting (FACS) analysis (Extended Data Fig. 8). Notably, while AIcrVIA1 and AIcrVIA2 were well tolerated in HEK293T cells, AIcrVIA3 exhibited toxicity (Extended Data Fig. 9). Together, these data demonstrate that AIcrVIAs can effectively suppress Cas13a-mediated RNA interference in mammalian cells, with AIcrVIA1 and AIcrVIA2 showing both potent activity and low toxicity supporting application as safe and precise regulators of RNA-targeting CRISPR–Cas systems in mammalian cells.

## Discussion

Acr proteins remain crucial for the post-translational regulation and control of CRISPR–Cas systems in gene-editing applications. To date, after over 10 years of research^42^, there are 118 experimentally validated Acrs targeting a diverse array of CRISPR–Cas systems^43^. However, the discovery of natural inhibitors remains challenging and time-consuming; even with cutting-edge developments in deep learning and structure-guided discovery, many effectors of interest still lack known Acrs^17–20^. Here we implemented a rapid approach to Acr design that leverages AI-driven protein fold generation to de novo create compact, potent and highly specific AIcrs. This approach is rapid, taking 8 weeks from target selection to verified nanomolar inhibition of CRISPR–Cas activity, which is much faster than traditional protein discovery workflows. Using this approach, we designed first in class AIcrVIAs targeted to the HEPN nuclease of LbuCas13a, a CRISPR–Cas system used for phage engineering and molecular diagnostics with no known Acrs^32,44,45^. The development of potent AIcrVIAs provides selectable markers for phage-engineering applications, akin to the application of phage-derived AcrVIA1 for phage engineering with *Listeria* *seeligeri* Cas13 (ref. ^27^). By leveraging targeted inhibitors, it may also be possible to fine-tune the interactions between phages and bacterial hosts or to better control Cas13 activity in human cells. Interestingly, like many naturally occurring Acrs^7^, our AIcrs exhibited no notable structural similarity to known proteins, a fact that limited attempts to structurally bootstrap toward naturally encoded Acrs. While the designs were not predictive of natural occurring type VI Acrs, their ability to potently inhibit LbuCas13a suggests the possibility that phages may have evolved HEPN inhibitors. With a larger sample size of AI-designed proteins to extract key interaction interfaces, it may be possible to discover Acrs similar to recent work identifying plant and fungal pathogen interactions^46^. Overall, our application of de novo protein design to generate AIcrs represents a powerful approach to overcoming the limitations of traditional discovery-based approaches.

While the discovery of naturally encoded Acrs yields mechanistically diverse inhibitors, approaches leveraging AI protein design afford user-defined control over the mode of inhibition (crRNA, DNA and RNA competition, active-site inhibition and allosteric inhibition). With an understanding of functionally relevant target sites, AI-driven protein design approaches could be applied to design bespoke inhibitors of any CRISPR–Cas class, type or subtype, including those for which no Acr is known to exist. For example, AIcrs could be designed to inhibit CRISPR Cas1–Cas2 acquisition machinery to develop switchable molecular recorders^47^ or tailored to regulate emerging classes of editors including TnpB, IsrB, IscB, Fanzor and the numerous CRISPR–Cas-guided DNA integrases^48–50^. In line with this, a very recent study described the development of BindCraft, an AI-driven approach to protein design with a demonstrated capability to design inhibitors of *Streptococcus* *pyogenes* Cas9, with applications in controlling its gene-editing activity^51^.

Beyond CRISPR–Cas, AI-driven protein design has greatly transformed in silico protein engineering, dramatically improving the efficiency of the design process and the success rate^29^. While revolutionary, the field is in its infancy and rapidly developing. At present, AI protein design outcomes remain difficult to predict. Retraining on the increasing volumes of wet-lab data will pave the way for a higher success rate in future, especially with the use of high-throughput quantitative cell-based screens. The development of one-shot protein design methods offers a tantalizing potential to overcome the need for screening a subset of designs^51^. However, target protein flexibility and conformational dynamics remain a notable challenge, especially in cases where structural insights may be limited. Furthermore, protein targets rarely exist in isolation; however, important advances in nucleic acid and cofactor interaction prediction using AlphaFold3 (ref. ^52^), Boltz-1 (ref. ^53^), Chai-1 (ref. ^54^) or RoseTTAFold All-Atom^55^ may overcome this challenge. It is likely that networks will need retraining on highly curated datasets to preserve the unique structural and functional properties of protein families. This approach contrasts with models trained on the PDB, which excel at generating robust proteins but may miss the nuanced features essential for the specificity and function of protein families. Tailored protein design will likely require a balance between the robustness provided by access to large training datasets and the precision afforded by training datasets tailored to specific protein families, functions or properties^56^. As the field rapidly accelerates, it seems inevitable that AI-driven protein design will have far-reaching impact across biological research.

## Methods

### Computational design and analysis of AIcrs

Lbu HEPN nuclease inhibitor design was carried out using RFdiffusion (version 1.1)^29^. A total of 10,000 70–130-aa designs of α and mixed αꞵ topology were generated against hotspots V411, V421, H473 and F995. Inverse folding was conducted using ProteinMPNN^30^, followed by energy minimization using FastRelax. Prediction quality was assessed by an AlphaFold2 initial guess implemented in ProteinMPNN. Designs were filtered by predicted aligned error (PAE) interaction scores < 10 (ref. ^59^). This subset of 80 designs underwent further rounds of ProteinMPNN (iterations or serial) to minimize PAE interact scores. The final set of 96 candidates were selected on the basis of manual AlphaFold2 model assessment, overall predicted local distance difference test and target r.m.s.d. Design diversity was assessed by MSTA in FoldMason^60^ (default parameters, GFP as root), visualized and annotated using iTol^61,62^. Multiple-sequence alignments were visualized using JalView^63^. Parameters describing design and target interactions (interface area (Å^2^), Δ*G*, *D*_max_, asphericity and radius of hydration) were calculated using the Python package MDAnalysis^64^ and PISA^65^. The combination of primary sequence and fold for the 96 selected binders was assessed using Foldseek^38^ online in 3Di/AA mode against six different databases: AlphaFold/UniProt50 version 4, AlphaFold/Swiss-Prot Database version 4, CATH50 version 4.3.0, MGnify-ESM30 version 1, PDB100 version 20240101 and GMGCL version 2204. For each alignment, we recorded the *E* value and visually inspected the matched PDB structure with high structural similarity to assess the extent of overlap. If the alignment corresponded only to a small local motif within a larger protein and did not match an autonomous protein fold or domain, the AIcr was considered to have no significant structural match. Each AIcr was *E*. *coli* codon-optimized and cloned into pET-29(+) with an in-frame C-terminal hexahistidine tag (Twist Bioscience). All crRNA, aRNA and reporter sequences used in vitro are listed in Supplementary Data 1.

### Expression and purification of Cas13 proteins

Cas13 homologs and their sequences are listed in Supplementary Table 2. Recombinant Cas13 was expressed as previously described^66^. Briefly, Cas13 was expressed in Terrific broth (TB) medium (with appropriate antibiotic), seeded with 0.5% (v/v) of overnight culture, incubated at 37 °C, 180 rpm until an optical density at 600 nm (OD_600_) of 0.5–0.6, cold-shocked for 20 min and induced with 0.5 mM IPTG followed by incubation at 16 °C, 180 rpm for 16 h. Cells were harvested by centrifugation (4,000*g* for 20 min) and flash-frozen in liquid nitrogen before storage at −80 °C. All buffers used to purify Cas13 homologs were the same except for the pH for TccCas13a (pH 7.5 throughout). Cells were resuspended in lysis buffer (50 mM Tris pH 7.0, 500 mM NaCl, 10 mM Imidazole, 5% (v/v) glycerol, 1 mM TCEP, 0.5 mM PMSF and EDTA-free protease inhibitor cocktail (Roche)) and lysed by sonication. The lysate was clarified by centrifugation (35,000*g*, 45 min at 4 °C) and the supernatant was incubated with Ni-NTA resin (Qiagen) for 1 h at 4 °C. The resin was washed with 10 CVs (column volumes) of buffer 1 (20 mM Tris pH 7.0, 500 mM KCl, 10 mM imidazole, 5% (v/v) glycerol and 1 mM TCEP), followed by 10 CVs of buffer 1 supplemented with 20 mM imidazole, followed by elution with 5 CVs of buffer 1 supplemented with 300 mM imidazole. Cas13 fractions were pooled and dialyzed overnight at 4 °C (14-kDa molecular weight cutoff (MWCO)) against 50 mM HEPES, 150 mM NaCl, 5% (v/v) glycerol and 1 mM TCEP in the presence of 1:50 weight ratio of tobacco etch virus protease if tagged with maltose-binding protein. The filtered dialysate was loaded onto a 5-ml HiTrap SP (Cytiva) and eluted over a 10-CV KCl gradient (0.15–1 M). Peak fractions were pooled and concentrated (30-kDa MWCO; Amicon) before SEC (S200 16/600, Cytiva), developed in 20 mM HEPES pH 7.0, 200 mM KCl, 10% (v/v) glycerol and 1 mM TCEP. Protein purity was assessed by SDS–PAGE and peak fractions were concentrated to 60 µM (30-kDa MWCO; Amicon). Purified protein was snap-frozen with liquid nitrogen in aliquots and stored at −80 °C.

### Cell-free AIcr production, screening and data analysis

Cas13a AIcr open reading frames were amplified with T7 forward and reverse primers (Supplementary Data 1) using 2× Q5 polymerase master mix (New England Biolabs) and purified with a QiaQuick PCR cleanup kit (Qiagen). Each AIcr was expressed from 2 nM of purified PCR product in PUREFrex 2.0 (GeneFrontier) cell-free expression system prepared in accordance with the manufacturer′s protocol. The reaction mixture was incubated for 4 h at 37 °C and held at 4 °C until assaying. LbuCas13a–crRNA ribonucleoprotein (RNP) complex (400 nM LbuCas13a and 400 nM crRNA (Integrated DNA Technologies) was prepared in 1× Cas13 cleavage buffer (20 mM HEPES–K pH 6.8, 50 mM KC, 5 mM MgCl_2_ and 5% (v/v) glycerol) at room temperature for at least 15 min and no longer than 1 h. AIcr and substrate reporter mix was prepared as 10 µl containing 0.67 µM fluorescence reporter RNA and 13.33 pM aRNA in 3× Cas13 cleavage buffer before the addition of 20 µl in vitro translated AIcr solution. Cas13a HEPN nuclease activity was assayed in black 384-well plates (Corning, 3820) by mixing 5 µl of RNP solution with 15 µl of AIcr–aRNA reporter solution. Fluorescence was immediately monitored every 1 min for 2 h at 37 °C (ClarioSTAR plus, BMG). The fluorescence values at 60 min were extracted and normalized against no-AIcrVIA controls to express as a fraction of activated LbuCas13a activity (heat map).

### AIcr small-scale protein expression and purification

AIcr plasmids were transformed into BL21 (DE3) *E*. *coli* and single colonies used to inoculate 2-ml cultures in overnight express instant TB medium (Novagen) containing 50 µg ml^−1^ kanamycin (Merck). Cultures were grown at 37 °C for 8 h, followed by 16 °C for 16 h, and centrifuged; cell pellets were frozen at −80 °C. Thawed cell pellets were chemically lysed (BPer, Thermo Fisher Scientific) for 20 min at room temperature and clarified by centrifugation at 17,000*g* for 10 min at 4 °C. Supernatant containing soluble AIcrs was incubated with Ni-NTA superflow resin (Qiagen) for 1 h at 4 °C and washed twice with buffer A (50 mM Tris pH 7.0, 500 mM NaCl, 5% (v/v) glycerol and 1 mM TCEP) containing 10 mM and 20 mM imidazole, respectively; proteins were eluted in buffer A containing 300 mM imidazole. Following batch purification, the concentration and purity of eluted AIcrs were confirmed using Coomassie-blue-stained SDS–PAGE analysis and QuickStart Bradford (Bio-Rad) assays.

### Cas13a–AIcr binding assays

Initial binding assessments were conducted using bilayer interferometry on a GatorPrime BLI instrument (Solve Scientific), with immobilized AIcrs to assess binding to recombinant LbuCas13 at 30 °C and shaking at 1,000 rpm throughout assay duration. LbuCas13a complexes were prepared at 5 µM with crRNA alone (1:1.2 molar ratio) or crRNA and aRNA (1:1.2:1.4 molar ratio) in 20 mM Tris-HCl pH 7.5, 150 mM KCl, 5% (v/v) glycerol and 1 mM TCEP and incubated for 10 min at room temperature, before dilution to 200 nM in Gator buffer Q (PBS pH 7.4, 0.2% (w/v) BSA and 0.02% (v/v) Tween 20). Batch-purified AIcrs were diluted 1:20 in buffer Q an immobilized on anti-His probes for 3 min, washed with buffer Q for 2 min, associated with prepared 200 nM Cas13 RNP complex for 3 min and dissociated in buffer Q for a further 3 min. All binding curves were baselined against ‘no-AIcr load′ runs. The maximum height of binding response normalized to AIcr load height response (all in nm) was used to generate the heat map.

### Cas13a–AIcrVIA competition assays and data analysis

In a bulk reaction, LbuCas13–crRNA RNP complex was prepared by complexing 133 nM LbuCas13a and crRNA (1:1) in 1× Cas13a cleavage buffer at room temperature for 15 min. The reaction was commenced by the addition of 15 μl of RNP complex into a black 384-well plate with 5 μL of aRNA reporter binder mix (prepared as above but with purified AIcrs) to give a final concentration of 1 μM AIcr, 100 nM RNP complex, 0.2 μM fluorescence reporter, 10 pM aRNA and 1× Cas13a cleavage buffer. The fluorescence output was measured on a plate reader (ClarioSTAR plus, BMG) for 2 h every 1 min at 37 °C.

For Cas13 AIcrVIA competition assays, 66.66 nM Cas13a RNP complex was assembled by mixing a 2:1 ratio of Cas13:crRNA at room temperature for 15 min in 1× Cas13a cleavage buffer. In a black 384-well plate, 15 μl of RNP complex was added to 5 μl of aRNA reporter binder mix to give a final concentration of 50 nM RNP complex, 1 μM–0.2 pM AIcr Cas13a candidates, 500 nM fluorescence reporter and various aRNA concentrations for different Cas13 homologs (LbuCas13a, 20 pM; LbaCas13a, 0.5 nM; TccCas13a, 1 nM; RfxCas13d, 50 nM; LbuCas13aΔ409–421, 50 pM) in 1× Cas13a cleavage buffer. The fluorescence output was measured on a plate reader every 30 s or 1 min for 30 min at 37 °C. Using GraphPad Prism version 10.1, the IC_50_ was calculated by measuring the linear slope of each reaction from 0 to 30 min for LbuCas13a, TccCas13a and RfxCas13d and from 0 to 15 min for LbuCas13aΔ409–421 and LbaCas13a. The slopes were analyzed with ‘inhibitor versus response ([three parameters)’ with constraints for the bottom set to 0 to generate IC_50_ values.

### Large-scale expression and purification of AIcrVIAs

AIcrVIA1–AIcrVIA3 sequences are listed in Supplementary Table 2 (Addgene, 234054, 234053 and 234052). *E*. *coli* BL21 (DE3) cells were transformed and cultured in TB medium supplemented with kanamycin (50 µg ml^−1^) at 37 °C, 200 rpm. At an OD_600_ of ~0.6, cultures underwent a cold shock and protein expression was induced with IPTG to a final concentration of 0.5 mM before incubating overnight at 18 °C. Cells were harvested by centrifugation at 4,000*g* for 25 min at 4 °C, washed with lysis buffer (20 mM Tris pH 7.5, 500 mM NaCl, 10% (v/v) glycerol and 10 mM imidazole), snap-frozen and stored at −80 °C. Cell pellets were resuspended in lysis buffer with 1 mM TCEP and EDTA-free protease inhibitor, at a ratio of 10 ml per 1 g of wet cell paste. Cells were lysed by sonication and lysates were clarified by centrifugation at 40,000*g* for 30 min at 4 °C. The clarified lysate was applied to Ni-NTA resin and incubated for 1 h at 4 °C with gentle agitation. The resin was subsequently washed with 10 CVs of lysis buffer with 20 mM imidazole and 1 mM TCEP before elution with 5 CVs of lysis buffer containing 200 mM imidazole and 1 mM TCEP. Fractions containing the protein of interest were pooled and concentrated using a concentrator (3-kDa MWCO; Amicon) before loading onto an S75 10/300 column (Cytiva) running in 20 mM Tris pH 7.5, 150 mM KCl, 5% (v/v) glycerol and 1 mM TCEP. Eluted protein fractions were either snap-frozen in liquid nitrogen or further concentrated using and stored at −80 °C until further purification. All three AIcrVIAs sequences were confirmed using liquid chromatography coupled to electrospray ionization tandem mass spectrometry.

### Fluorescence polarization

LbuCas13a–crRNA RNP complexes were assembled in 1× Cas13a cleavage buffer for 15 min at room temperature. RNP complexes were serially diluted twofold and incubated with 40 nM 5′6-FAM-labeled aRNA and 2 μM AIcrVIAs (final concentration) for 2 h at 37 °C. The fluorescence polarization and anisotropy values were measured every 5 min to determine whether the system was at equilibrium (ClarioStar plus, BMG LabTech). Fluorescence anisotropy and polarization values were calculated using MARS Data analysis (BMG LabTech) and the curve was fitted using one-site-specific binding (GraphPad Prism version 10.4).

### Circular dichroism

CD spectra were recorded using a J-810 CD Spectrometer (JASCO). Protein samples at a concentration of 0.3 mg ml^−1^ were prepared by dilution in water, resulting in a final buffer composition of 0.3 mM Tris pH 7.5, 2.5 mM KCl and 0.08% (v/v) glycerol. Triplicate wavelength scans were recorded from 260 to 190 nm at 30 °C. For thermal denaturation experiments, protein samples (0.3 mg ml^−1^) were heated from 30 °C to 70 °C to 30 °C. Wavelength scans from 260 to 190 nm were recorded at intervals of 10 °C, following a 5-min incubation period at each target temperature. The recorded data were averaged, baseline-corrected and smoothed using JASCO spectra manager version 2. Data were further analyzed on GraphPad Prism version 10.4 using the data recorded between 195 and 260 nm.

### Crystallization and structure determination of AIcrVIA1

AIcrVIA1 was crystallized using the hanging-drop vapor diffusion method at 10 mg ml^−1^ in 0.1 M Bis–Tris propane (pH 7.2) and 2.6 M ammonium sulfate at room temperature. Crystals were were isolated after 10 days and flash-frozen directly into liquid nitrogen without cryoprotectant. X-ray diffraction data were recorded on a Dectris Eiger 16M detector at the MX2 beamline of the Australian Synchrotron. Data reduction was performed using XDS^67^. The initial atomic model was obtained by molecular replacement in PHENIX (version 1.20.1)^68^ using the structure of AIcrVIA1 predicted by AlphaFold2 (ref. ^69^). The model was manually adjusted in WinCoot (version 0.9.8.93)^70^ to fit the electron density map and iteratively refined using phenix.refine. The model and data were deposited to the PDB under accession code 9MVR. All figures and visualizations were generated using ChimeraX.

### Thermal shift assay

All thermal shift assays were conducted using the QuantStudio 6 and 7 Flex real-time PCR systems (Thermo Fisher Scientific) with SYPRO protein dye (Invitrogen). In each well of a MicroAmp fast optical 96-well reaction plate (Applied Biosystems), 770 nM LbuCas13a was mixed with a 1:2 molar ratio of crRNA in 1× cleavage buffer and complexed at room temperature for 15 min. Subsequently, aRNA was added in a 1:4 molar ratio, followed by a 1:1 molar ratio of AIcrsVIAs, and the mixture incubated for 15 min before the addition of 2.5 μl of 40× SYPRO orange to each well. Fluorescence changes were monitored from 25 °C to 95 °C at a rate of 0.1 °C s^−1^ and the normalized relative fluorescence units (RFU) were plotted against the temperature (*n* = 3 technical replicates).

### Cryo-EM sample preparation and data collection

The LbuCas13–crRNA–aRNA–AIcrVIA1 complex was assembled in cryo-EM buffer (20 mM Tris pH 7.0, 30 mM KCl, 5 mM MgCl_2_ and 2% (v/v) glycerol). LbuCas13a was combined with crRNA, aRNA and AIcrVIA in a molar ratio of 1:1.2:1.4:5, respectively. Assembly was carried out sequentially with a 5-min incubation at room temperature after each addition to facilitate complex formation. Subsequently, 100 µl of the assembled complex solution was purified and fractionated over a S200 Increase 3.2/300 SEC column (in cryo-EM buffer) attached to an AKTAMicro (Cytiva). The purity and composition of fractions were verified using SDS–PAGE and 12% (w/v) urea–PAGE. The resulting complex was immediately used to prepare cryo-EM grids by applying 3.5 µl onto amylamine glow-discharged UltrAufoil R1.2/1.3, 300-mesh gold grids (Quantifoil) before blotting with a force of −3 for 3 s under 100% humidity at 4 °C (Vitrobot Mark IV, Thermo Fisher Scientific) and plunge-freezing in liquid ethane. Cryo-EM data were collected using a Talos transmission EM instrument (Thermo Fisher Scientific) operating at 200 kV. A total of 2,544 videos were recorded at a calibrated pixel size of 0.93 Å^2^ per pixel over 50 frames with an exposure of 1 e^−^ per Å^2^ per frame, resulting in a total electron dose of 50 e^−^ per Å^2^. The defocus range was set between −0.8 and −1.4 µm to optimize image contrast and resolution.

### Cryo-EM data processing and model building

Dose-fractionated frames were corrected for beam-induced motion and radiation damage using MotionCor2 (version 1.4.5)^71^. The aligned, dose-weighted averages were then imported into cryoSPARC (version 4.5.2) for further processing. Contrast transfer function parameters were estimated with CTFFIND (version 4.1.14)^72^. Micrographs were curated at a resolution of 5 Å or higher. Template picking was prepared by performing blob picking in cryoSPARC (version 4.5.2)^73^, followed by five rounds of two-dimensional classification and heterogeneous refinement. Particles selected from the highest-quality heterogeneous refinement density map were used to train a Topaz 0.2.5 model. All particles picked using Topaz (version 0.2.5)^74,75^ were extracted in a 256 × 256-pixel box before heterogeneous refinement and ab initio reconstruction. The resulting particle stack underwent three rounds of iterative three-dimensional (3D) classification and refinement using cryoSPARC nonuniform refinement^76^. Conformational flexibility and heterogeneity in the final particle stack (20,357 particles) were investigated using cryoSPARC 3D variability analysis^77^ (resolution filter of 8 Å, three components). The series with the most extensive and continuous movement of AIcrVIA1 was visualized using ChimeraX over 20 frames.

### Model building

The structure of the LbuCas13a–crRNA–aRNA–AIcrVIA1 complex was built from the previously published LbuCas13a–crRNA–aRNA structure (PDB 5XWP) and the X-ray crystal structure of AIcrVIA1 described in this study. The initial model was fit to the density map in ChimeraX before iterative phenix.refine^68^ (version 1.18.2; rigid-body fitting, global minimization and atomic displacement parameter refinement restrained with secondary-structure restraints and AIcrVIA1 reference model restraints) and real space refinement in Coot (version 0.9.4^70^). The model and data were deposited to the PDB under accession code 9MVS. All figures and visualizations were generated using ChimeraX.

### Phage propagation and phage restriction assays

T4 phage were propagated in *E*. *coli* B strains in Luria–Bertani (LB) medium. Briefly, 100 µl of overnight *E*. *coli* culture was inoculated in 10 ml of fresh medium and supplemented with 10 mM MgSO_4_ and 10 mM CaCl_2_. The culture was grown until log phase (OD_600_ of 0.5) before infection with 100 µl of ~10^8^ plaque-forming units (PFU) per ml of T4 phage lysate and incubating overnight at 37 °C shaking. The overnight culture was centrifuged 4000*g* and the cleared cell lysate was treated with 10% (v/v) chloroform to lyse any remaining cells before further centrifugation. The supernatant was passed through a 0.22-µm filter to obtain pure phage lysate.

All phage plaque assays were carried out in *E*. *coli* DH10B (New England Biolabs) using the double-layer top agar method. For the binder inhibition assays, DH10B cells were transformed with plasmids carrying an aTc-inducible pTET-LbuCas13a (ref. ^32^) with a coexpressing crRNA targeting T4 *soc* and an arabinose-inducible pBAD vector carrying the binder protein or an empty vector. Cloning was performed using In-Fusion cloning (Takara, 638947) through PCR amplification of both the vector backbone and the insert, followed by recombination with the In-Fusion master mix according to the manufacturer’s instructions. The primers used are listed in Supplementary Data 1. The strains were grown overnight in LB medium containing 100 µg ml^−1^ ampicillin and 35 µg ml^−1^ chloramphenicol. A 100-µl aliquot of overnight culture was inoculated into fresh Lennox medium and induced at log phase with 2.5 nM aTc ± 2% arabinose to induce the expression of the Cas13 effector and binders, respectively. Induced culture was incubated overnight at 37 °C shaking. The following day, soft Lennox agar (0.5% (w/v) agar) supplemented with 1 ml of overnight culture was poured onto Lennox agar plates supplemented with antibiotics and inducers. Phage titers were calculated by spotting 3-µl drops of serially diluted phage lysate (10^9^–10^1^ PFU/mL) and incubated overnight at 37 °C. Plaques were counted after incubation and represented as PFU per ml. Where plaque formation was inhibited, a single plaque was counted on the first dilution with no plaques. Biological triplicates were performed for all binders and the efficiency of plaquing (EOP) in their presence was plotted.

All liquid phage restriction inhibition experiments were performed in a Tecan Infinite M200 plate reader. Briefly, *E*. *coli* DH10B strains carrying pTET-LbuCas13a with a coexpressing crRNA and pBAD vector carrying the binder protein (Addgene, 231127, 231126 and 231125) were grown overnight at 37 °C with appropriate antibiotics. Then, 100 µl of overnight culture was subcultured into fresh Lennox medium and induced at log phase with 2.5 nM aTc ± 2% arabinose to induce the expression of the Cas effector and binders. Induced culture was incubated overnight at 37 °C. Overnight culture was then added at 1:100 dilution to a 96-well plate and monitored on the plate reader until it reached an OD of 0.1. Wells were then infected with T4 phage at a multiplicity of infection of 0.0005 and incubated while shaking for 16 h, collecting OD_600_ readings every 20 min. Biological triplicates of all assays were performed and data were plotted using GraphPad Prism version 10. The schematics were created with BioRender.com.

### Human cell culture

The HEK293T cell line (American Type Culture Collection, CRL-3216) was cultured in high-glucose DMEM (Thermo Fisher, 11965092) containing 10% heat-inactivated FBS (Thermo Fisher, 10100147), 100 mg ml^−1^ penicillin–streptomycin (Thermo Fisher, 151401220) and 2 mM GlutaMAX (Thermo Fisher, A1286001). HEK293T cells were maintained at confluency between 20% and 80% in 37 °C incubators with 10% CO_2_.

### Silencing assay

All primers, gBlock templates and crRNA sequences are listed in Supplementary Data 1. The crRNA (U6 promoter) and eGFP (EF1α promoter) were coencoded on plasmid pRDA_221 (Addgene, 169142; gifts from J. Doench and D. Root). crRNA PCR amplicons were inserted into pRDA_221 between AgeI and EcoRI restriction sites using Gibson assembly. Human codon-optimized AIcr genes were amplified by PCR and cloned into pLX_TRC311 using In-Fusion assembly (Takara, 638947). The pLX_TRC311 vector (Addgene, 113669; gift from J. Doench) drives expression from the EF1α promoter. NLS–LbuCas13a–NLS–3HA was expressed from a lentiCRISPRv2 backbone, also under EF1α promoter control. The LbuCas13a expression construct was generated by Gibson assembly of synthetic gBlocks (Integrated DNA Technologies) into lentiCRISPRv2-Blast (Addgene, 98293; gift from B. Stringer) between AgeI and MluI restriction sites. To monitor collateral cleavage activity, mCherry was expressed from a pMSCV-IRES-mCherry FP plasmid. This plasmid was a gift from D. Vignali (Addgene, plasmid 52114; RRID:Addgene_52114).

All DNA transfection experiments were performed using an optimized Lipofectamine 3000 transfection protocol (Thermo Fisher, L3000015). Cells were transfected in 24-well plates when they reached 70–90% confluency. For each well containing 500 μl of medium, 500–600 ng of DNA plasmids were mixed with 1 μl of P3000 reagent and 1.5 μl of Lipofectamine 3000 in Opti-MEM serum-free medium (Thermo Fisher, 31985070) to a total of 53 μl. Two targeting crRNAs with the highest on-target cleavage were chosen and compared to one nontargeting crRNA as the negative control to establish relative silencing efficiency. The plasmid mixture was incubated for 15 min at room temperature and then added to each well for efficient delivery of plasmids (crRNA tests: 350 ng of LbuCas13–NLS–3HA + 250 ng of crRNA; AIcr experiment: 250 ng of LbuCas13 + 150 ng of crRNA + 100 ng mCherry + 100 ng AIcr; control experiments: 250 ng of RfxCas13d + 150 ng of crRNA + 100 ng of mCherry + 100 ng of AIcrs). Transfected cells were incubated at 37 °C and 10% CO_2_ and the transfection efficacy was monitored 24 and 48 h after transfection by fluorescece microscopy. Fluorescence images were taken 48 h after transfection.

### Cell flow cytometry

The cells were resuspended in 100 µl of PBS with 2% FBS (v/v) for flow cytometry analysis. Flow cytometry analysis was performed using the FACS Symphony Cell Analyzer A5 (BD Biosciences). All flow cytometry profiles were analyzed using FlowJo version 10 software (Tree Star).

### Western blot

Cells were washed three times with ice-cold PBS and lysed on ice in RIPA lysis buffer (50 mM Tris (Sigma-Aldrich, T1530) pH 8.0, 150 mM NaCl, 1% NP-40 (Sigma-Aldrich, I18896), 0.1% SDS and 0.5% sodium deoxycholate (Sigma-Aldrich, D6750)) containing protease inhibitor cocktail (Roche, 04693159001) and phosphatase inhibitor cocktail (Roche, 4906845001). Samples were incubated for 30 min at 4 °C with rotation (25 rpm) and centrifuged at 16,000*g* for 10 min at 4 °C. Supernatant was transferred to a new tube. Protein concentrations were quantified using the Pierce BCA protein assay kit (Thermo Fisher, 23225) according to the manufacturer’s instructions. A total of 10 μg of protein diluted in 1× Bolt LDS sample buffer (Thermo Fisher, B007) and 1× Bolt sample reducing agent (Thermo Fisher, B009) was denatured at 95 °C for 5 min. Samples were resolved by Bolt Bis–Tris Plus 4–12% gels (Thermo Fisher, NW04120BOX) in 1× MES SDS running buffer (Thermo Fisher, B0002) and transferred to 0.45 μM PVDF membranes (Thermo Fisher, 88518) by a Trans-Blot semidry electrophoretic transfer cell (Bio-Rad) at 20 V for 30 min. Membranes were incubated in blocking buffer 5% (w/v) BSA (Sigma-Aldrich, A3059) in Tris-buffered saline with 0.15% Tween 20 (TBST; Sigma-Aldrich, P1379) for 1 h at room temperature and probed overnight with primary antibodies at 4 °C. Blots were washed three times in TBST, followed by incubation with fluorophore-conjugated or horseradish peroxidase (HRP)-conjugated secondary antibodies for 1 h at room temperature. Membranes were washed in TBST three times and chemiluminescence was detected using the ChemiDoc Imaging System (Bio-Rad). The following antibodies were used: mouse anti-HA monoclonal antibody (Cell Signaling, 2367; 1:2,000 dilution), mouse anti-β-actin monoclonal antibody (Sigma-Aldrich, A2228; 1:10,000 dilution) and rabbit anti-mouse IgG–HRP (Dako, P0260; 1:10,000 dilution).

### Fluorescence microscopy analysis

For RNA silencing experiments, the fluorescence intensity was monitored using an EVOS M5000 FL cell imaging system (Thermo Fisher). From each well, 4–6 images were taken 48 h after transfection. The fluorescence intensity of each image was quantified using a lab-written macro in ImageJ software. Briefly, all images obtained from a single experiment are simultaneously processed using a batch-mode macro. First, images were converted to 8 bits, threshold-adjusted and converted to black and white using the ‘convert to mask’ function; the fluorescence intensity per pixel was measured using the ‘analyze particles’ function. Each single mean fluorescence intensity was obtained from six different fields of view for each crRNA and subsequently normalized to the nontargeting control crRNA. A twofold or higher reduction in fluorescence intensity was considered biologically relevant. Data analyses were performed in GraphPad Prism version 9.

### Reporting summary

Further information on research design is available in the Nature Portfolio Reporting Summary linked to this article.

## Online content

Any methods, additional references, Nature Portfolio reporting summaries, source data, extended data, supplementary information, acknowledgements, peer review information; details of author contributions and competing interests; and statements of data and code availability are available at 10.1038/s41589-025-02136-3.

## Supplementary information

Supplementary Information Supplementary Figs. 1–14, Tables 1 and 2 and Source data for Supplementary Figs. 10 and 13.

Reporting Summary

Supplementary Data 1 List of DNA and RNA oligonucleotides (three tables).

Supplementary Video 1 A 3D variability analysis of the AIcrVIA1–Cas13 complex.

## Source data

Source Data Fig. 3 Raw activity data for 96 binder designs (semipurified and cell-free expression; two tables).

Source Data Extended Data Fig. 3 Unprocessed and uncropped urea gels and SDS–PAGE.

Source Data Extended Data Fig. 5 Unprocessed and uncropped SDS–PAGE.

Source Data Extended Data Fig. 9 Unprocessed and uncropped western blots.

## Figures and Tables

**Extended Data Table 1:**
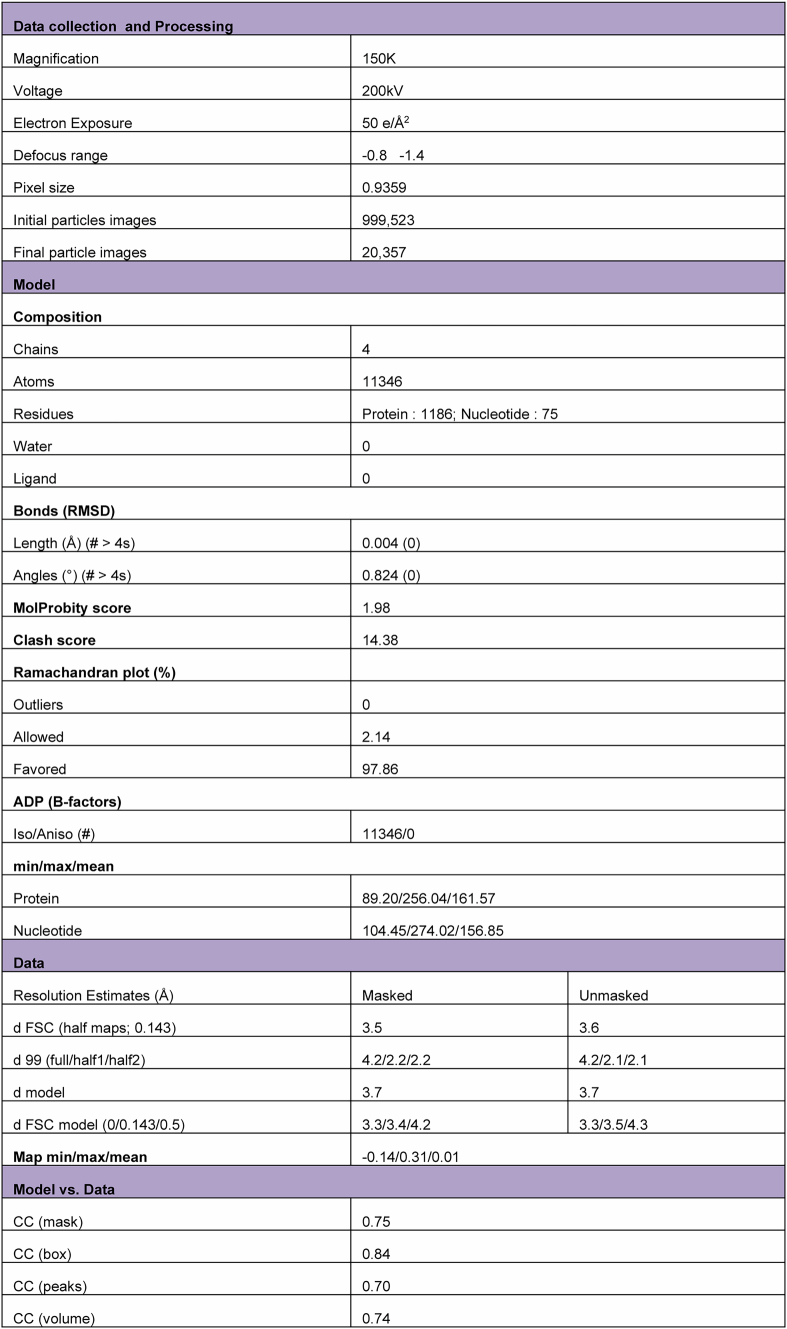
Taveneau et al. leverage AI-driven protein design to create inhibitors that control RNA-targeting enzymes in cells, revealing a strategy to rapidly design off-switches for RNA editing systems

## Data Availability

The cryo-EM and X-ray crystallography structures were deposited in the PDB under accession codes 9MVS (cryo-EM) and 9MVR (X-ray). Plasmids encoding AIcrVIA1–AIcrVIA3 were deposited to Addgene (pBAD vectors carrying the binder proteins: 231127, 231126 and 231125; pET29b+ vectors: 234054, 234053 and 234052). Source data are provided with this paper.

## Code availability

No new or custom code was generated as part of this research.
