# Supplementary material for: De novo design of potent CRISPR–Cas13 inhibitors
Source: Nat Chem Biol. 2026 Jan 26;22(8):1342–50. doi: 10.1038/s41589-025-02136-3 (PMC13423800; doi:10.1038/s41589-025-02136-3)

nature chemical biology

Supplementary information https://doi.org/10.1038/s41589-025-02136-3

De novo design of potent CRISPR–Cas13 inhibitors

In the format provided by the authors and unedited

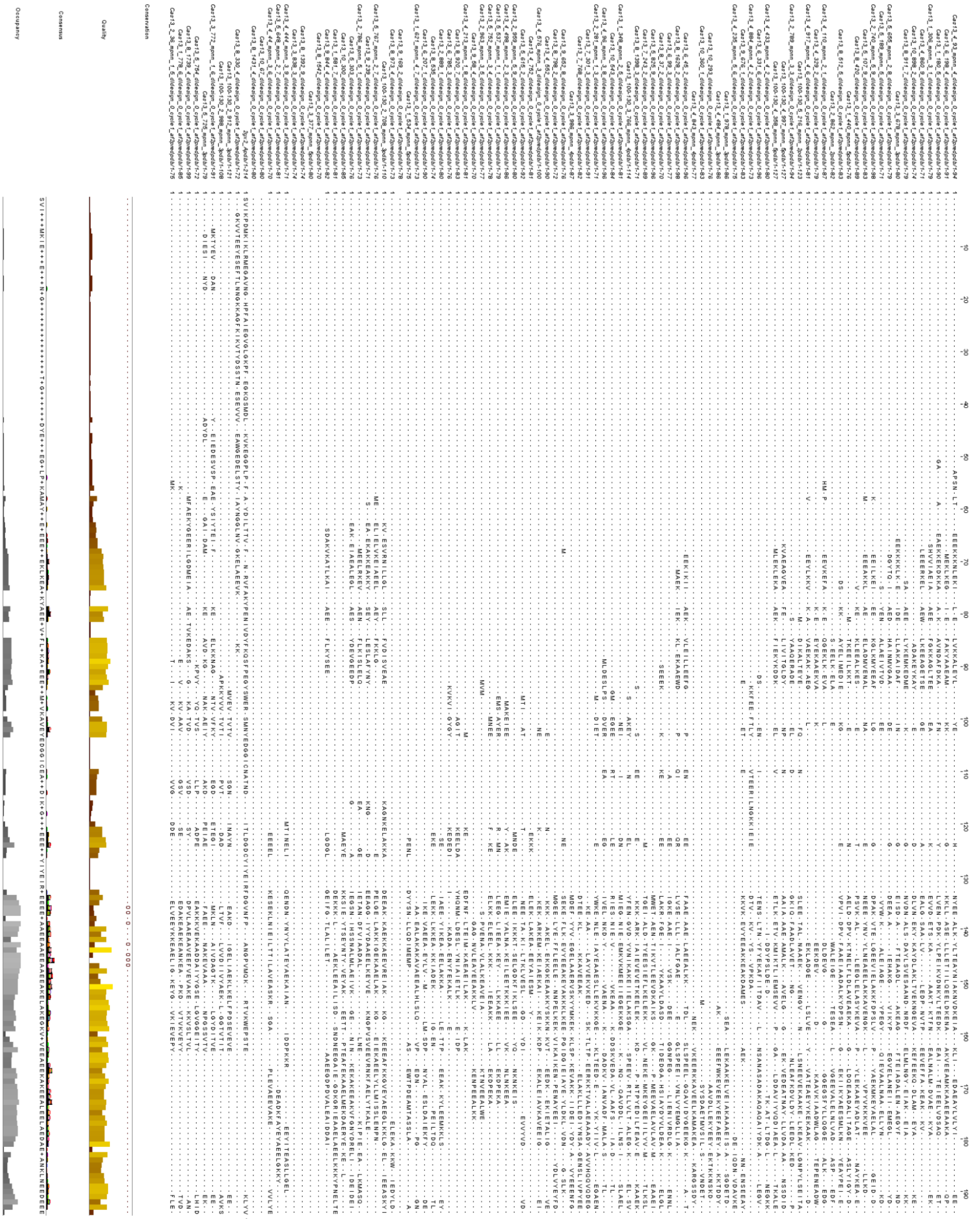

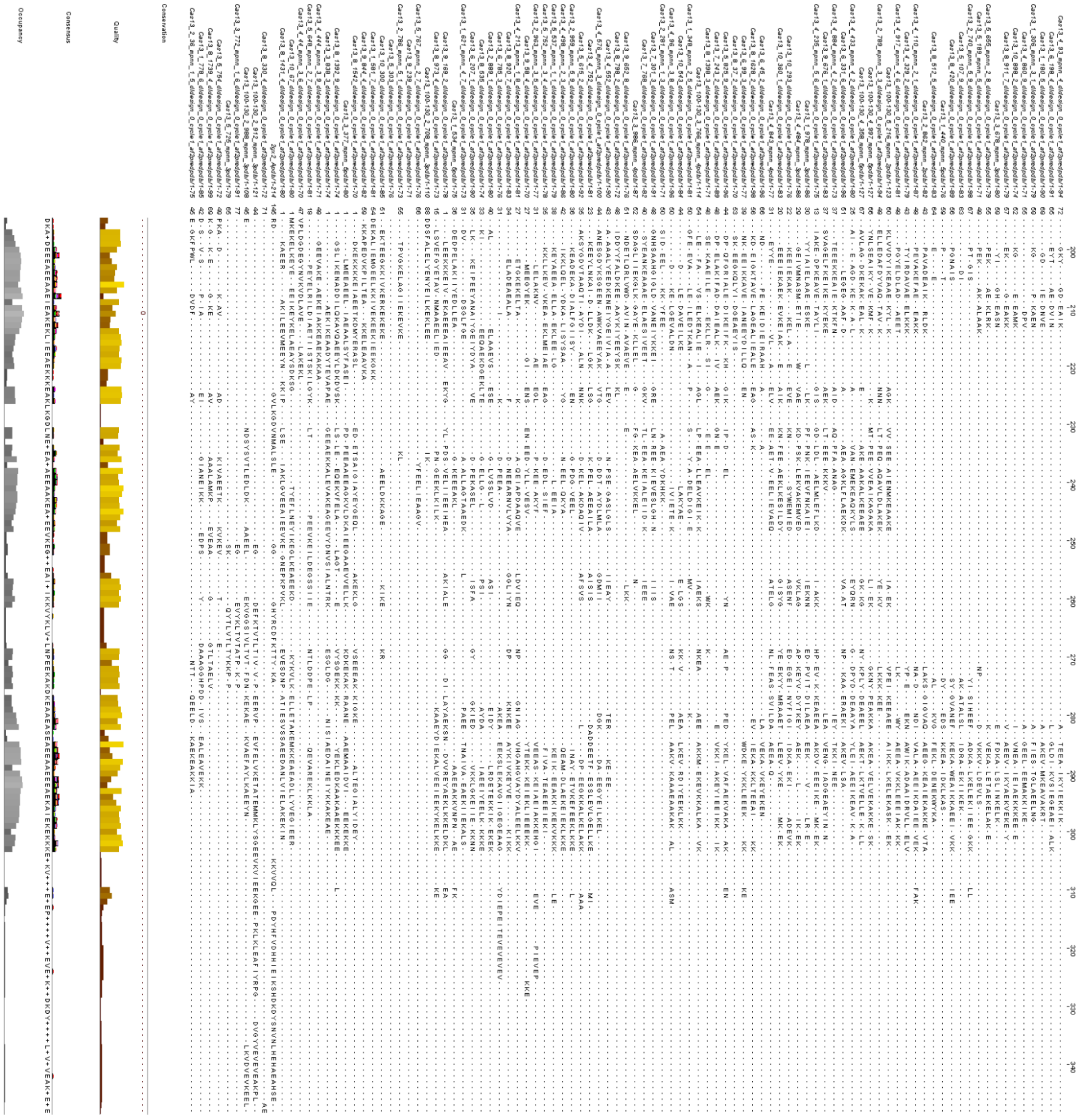

Supplementary Figure 1. Multiple sequence alignment used for multiple structure alignment (MSTA). Multiple sequence alignment was generated using FoldMason[60] and visualized using JalView[63], integrating both structural alignment and primary amino acid sequence data from 96 AIcr candidates selected for binding and inhibition of LbuCas13a. The combined structural and sequence-based approach revealed no conserved placement of amino acids in primary sequence and matches the diverse structural landscape.

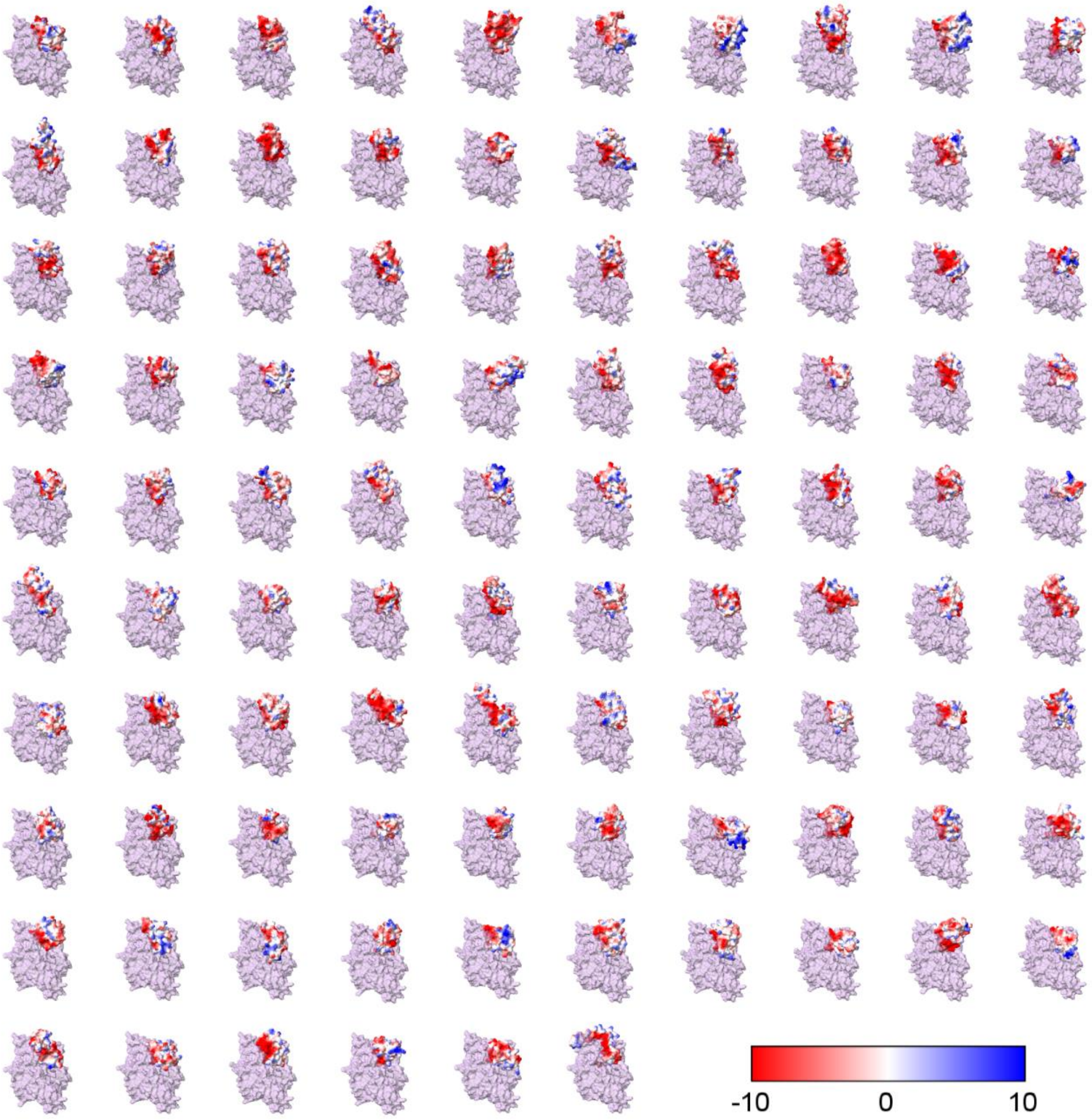

Supplementary Figure 2. Charge complementarity between AIcr and LbuCas13a active site, mimicking RNA chemical properties. Coulombic electrostatic surface potential (ChimeraX) of each of the 96 AIcrs (red-blue, surface) in interaction with the HEPN domains of LbuCas13a (surface, purple) used as an input in RF-Diffusion (bottom, transparent). Most designs are predicted to interact via an acidic patch directly with the HEPN domain via charge complementarity.

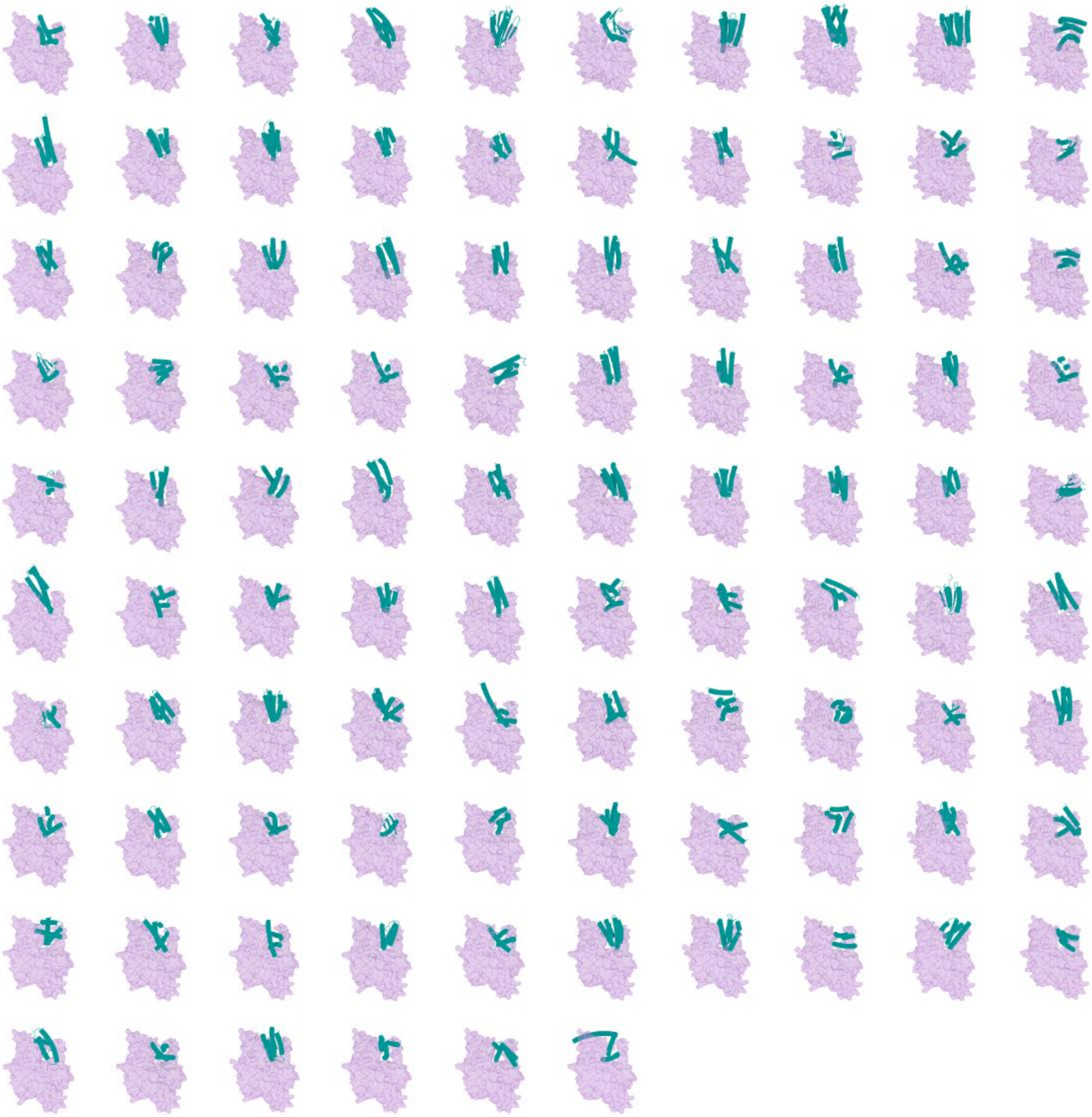

Supplementary Figure 3. AIcr designs are predicted to interact with the active site of LbuCas13a. The HEPN domains of LbuCas13a (surface, purple), used as an input in RF-Diffusion, along with each of the 96 different in silico-designed AIcrs (cartoons, teal).

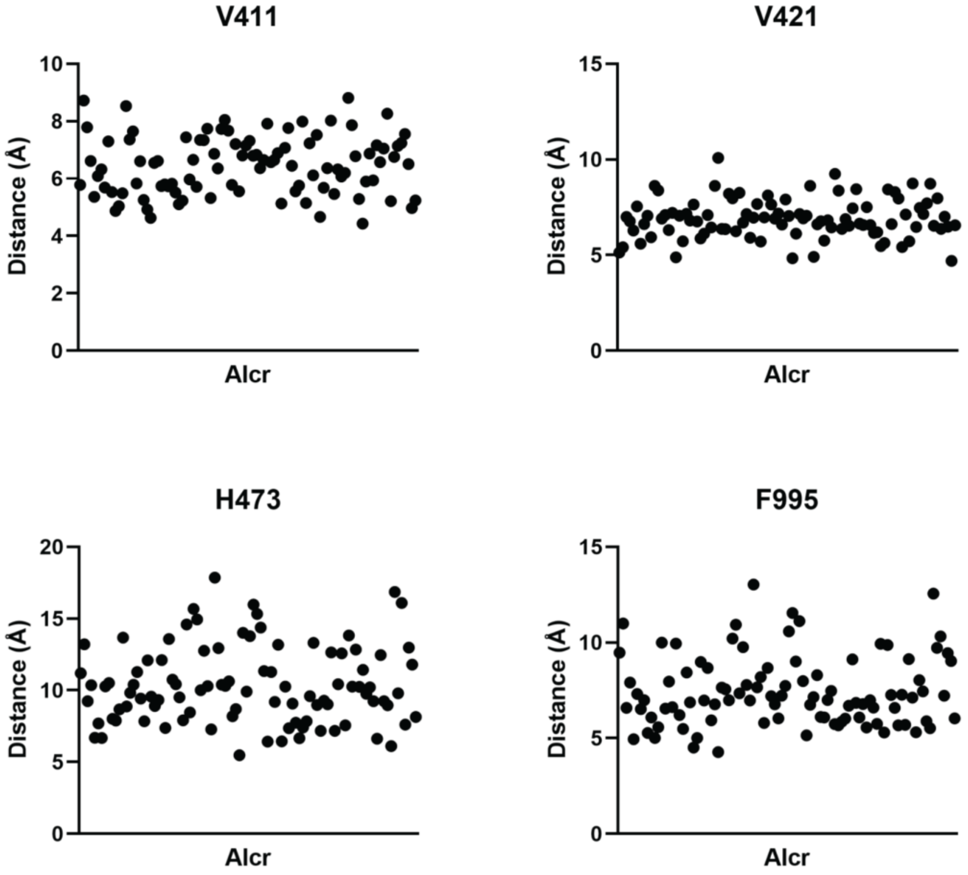

Supplementary Figure 4. Distance between key hotspot residues of LbuCas13a and the 96 potential AIcr designs. Minimum distances between the key hotspot residues used for the AIcrs design (V411, V421, H473, and F995) in the LbuCas13a active site and each of the 96 AIcr designs. Most AIcrs are predicted to be between 5 – 15 Å, reflecting potential interactions at the LbuCas13a active site.

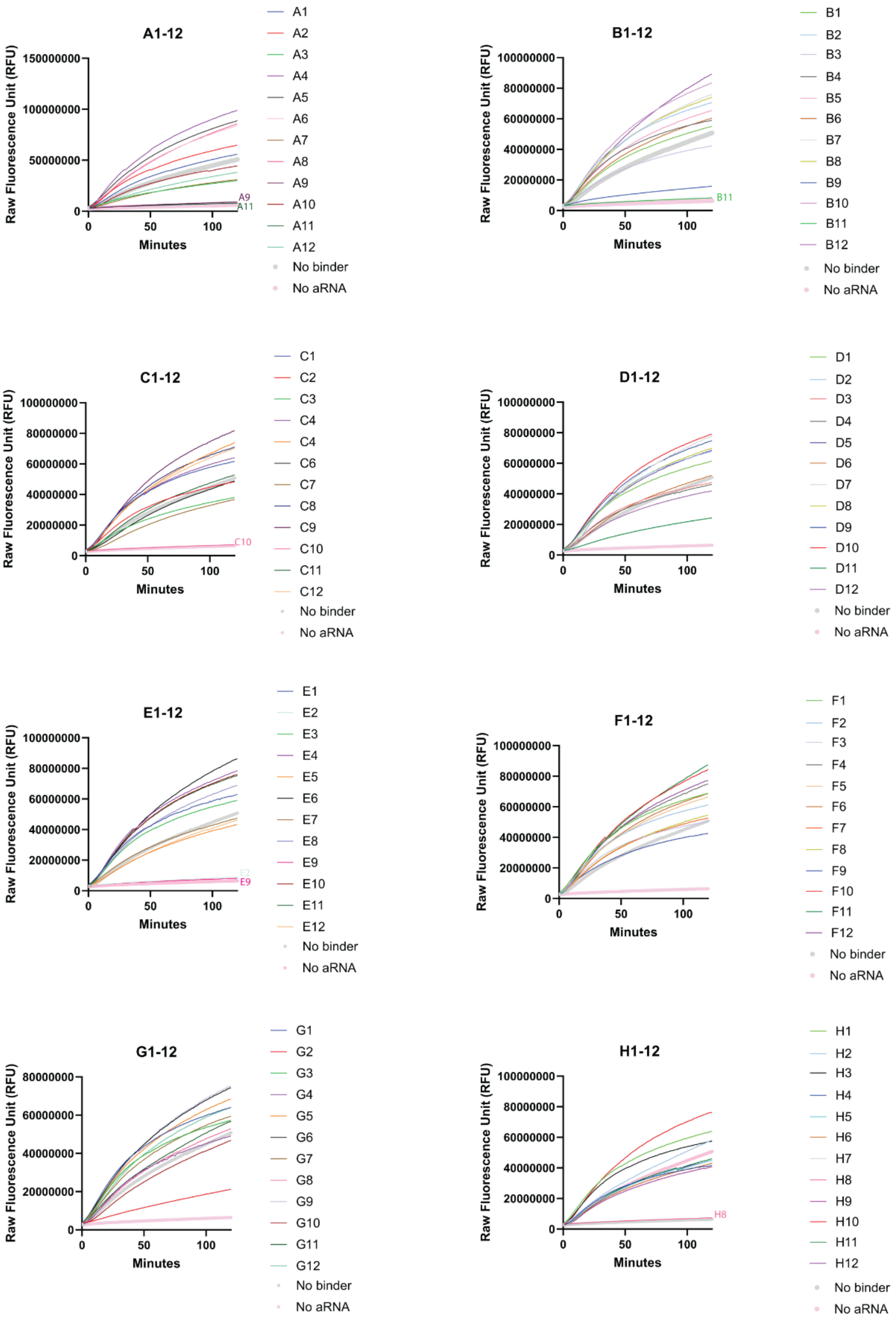

Supplementary Figure 5. LbuCas13a HEPN nuclease activity assay in the presence of cell-free expressed AIcrs. Each AIcr (A1-H12 in a 96 well plate) was assayed with LbuCas13a-crRNA-aRNA in the presence of labelled reporter RNA. Activity from LbuCas13a leads to increased fluorescence (RFU) over time (mins).

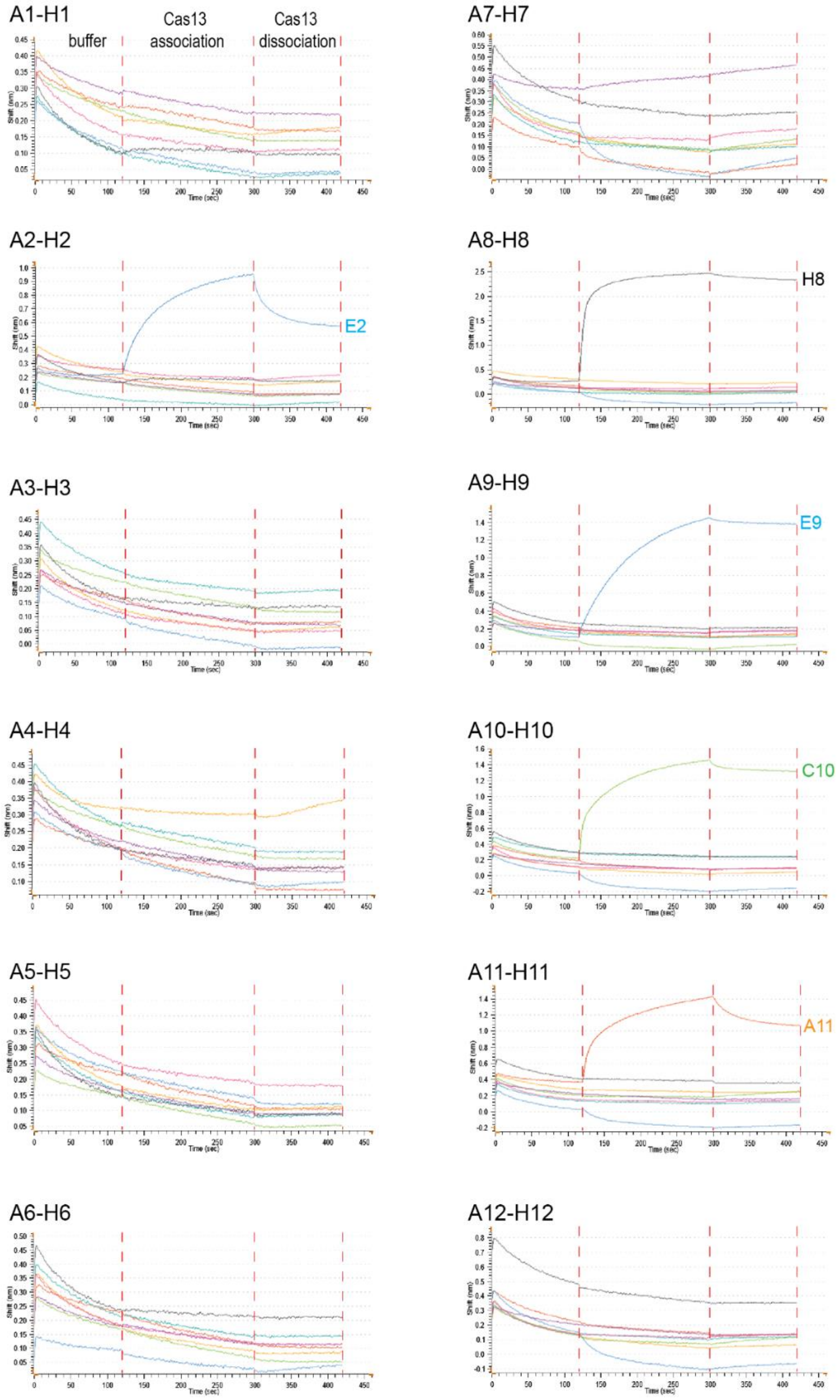

Supplementary Figure 6. Raw biolayer interferometry data for AIcr binding to the LbuCas13a-crRNA complex. Each panel represents data from a plate column containing eight AIcr designs (A1-H1, A2-H2, etc.) loaded onto Anti-His probes, followed by a wash with buffer Q. The association phase shows the binding of LbuCas13a-crRNA to AIcr, while the dissociation phase follows after an additional wash with buffer Q. The signals were normalized by subtracting the data from a 'no AIcr bound' control experiment using the same probes. The difference in signal is shown for the wash, association, and dissociation phases.

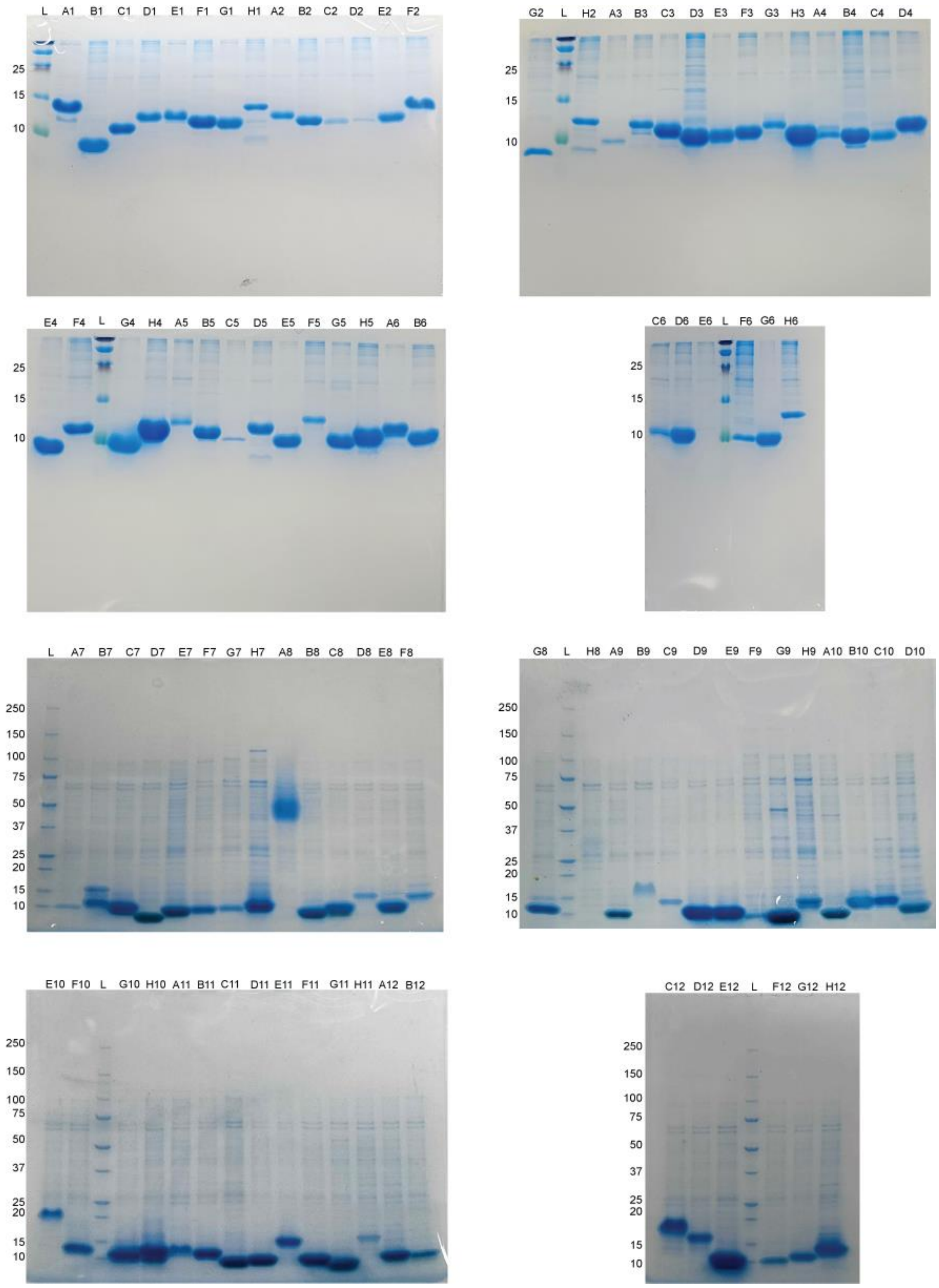

Supplementary Figure 7. Small-scale purification of AIcrs. AIcrs were expressed and purified in small scale followed by batch-based affinity chromatography and SDS-PAGE. (TOP) 12% (w/v) SDS-PAGE gels, (BOTTOM) 4-20% (w/v) gradient SDS-PAGE gels. The ladder (L) molecular weights (MW) are in kDa (left side of gel).

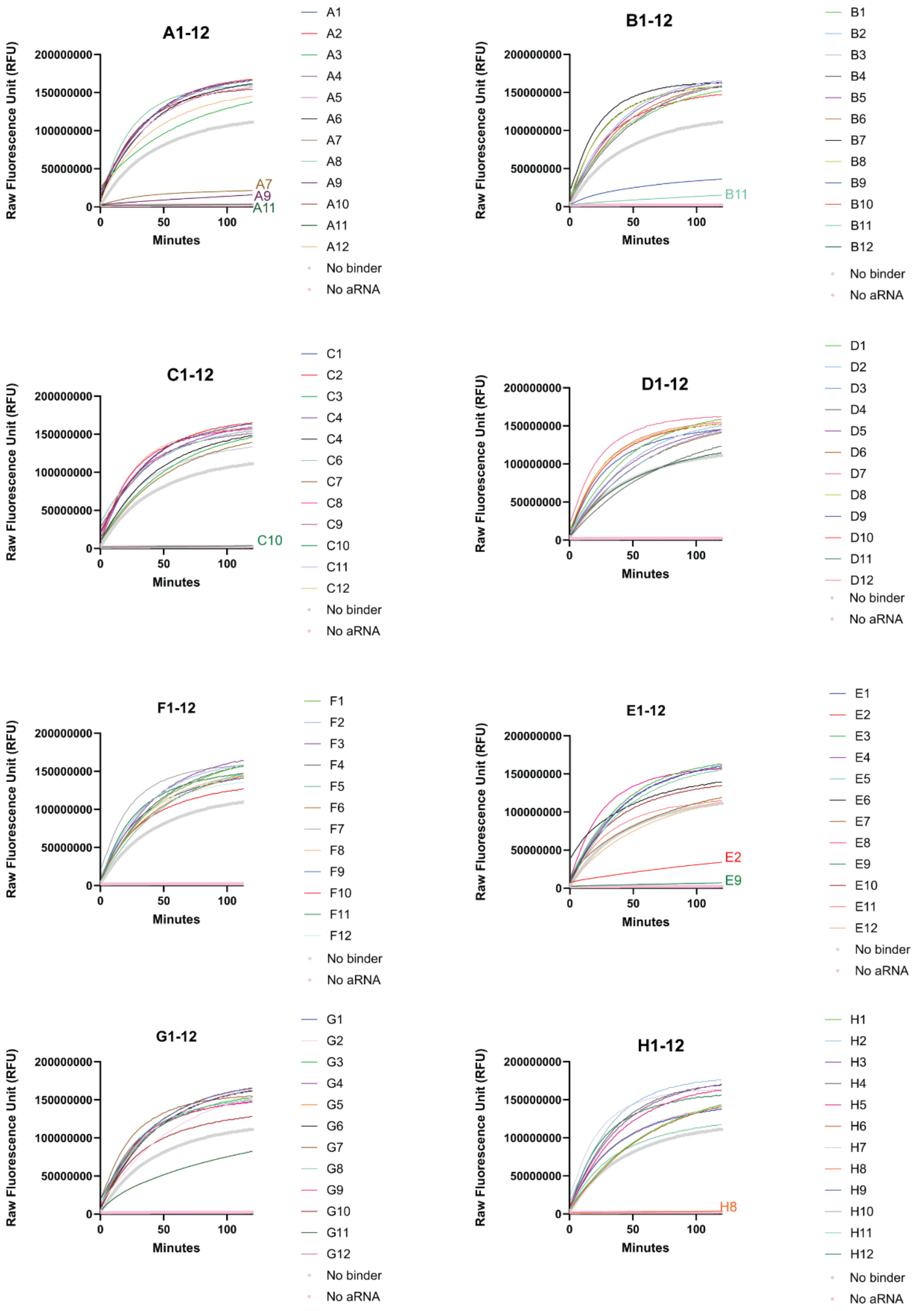

Supplementary Figure 8. LbuCas13a HEPN nuclease activity assay in the presence of semi-purified AIcrs. Each AIcr (A1-H12 in a 96 well plate) was assayed with LbuCas13a-crRNA-aRNA in the presence of labelled reporter RNA. Activity from LbuCas13a leads to increased fluorescence (RFU) over time (mins).

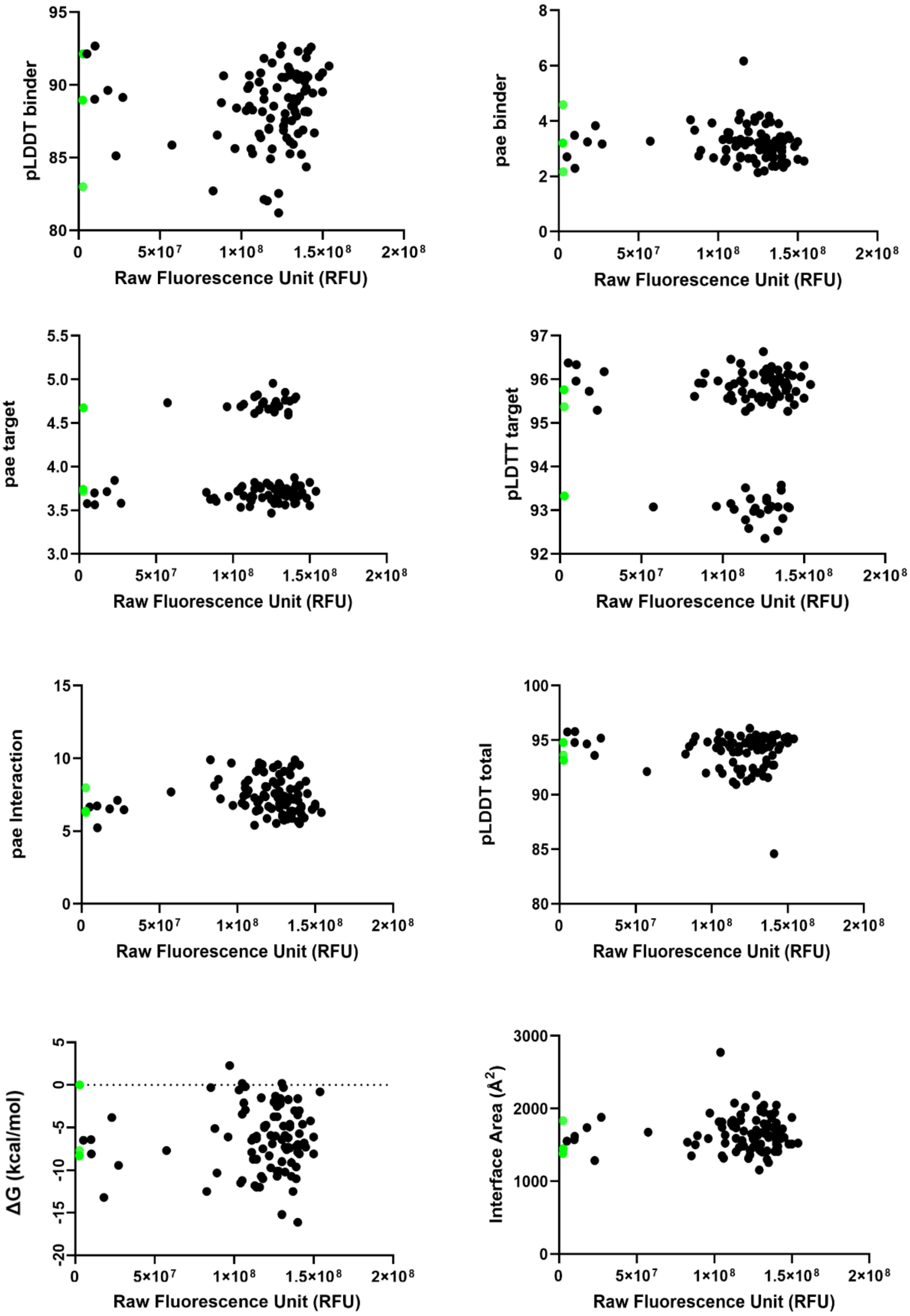

Supplementary Figure 9. Comparative analysis of PAE, pLDDT scores, predicted ΔG, and interaction surface area. Correlation analysis of these parameters with LbuCas13a-crRNA-aRNA activity in the presence of 96 AIcrVIA variants. Data points representing AIcrVIA1, VIA2, and VIA3 are highlighted in green. No correlation was observed between the inhibition capacity of AIcrVIA variants and any of these parameters (PAE, pLDDT scores from AF2 initial guess; predicted ΔG and interaction surface area calculated using PISA).

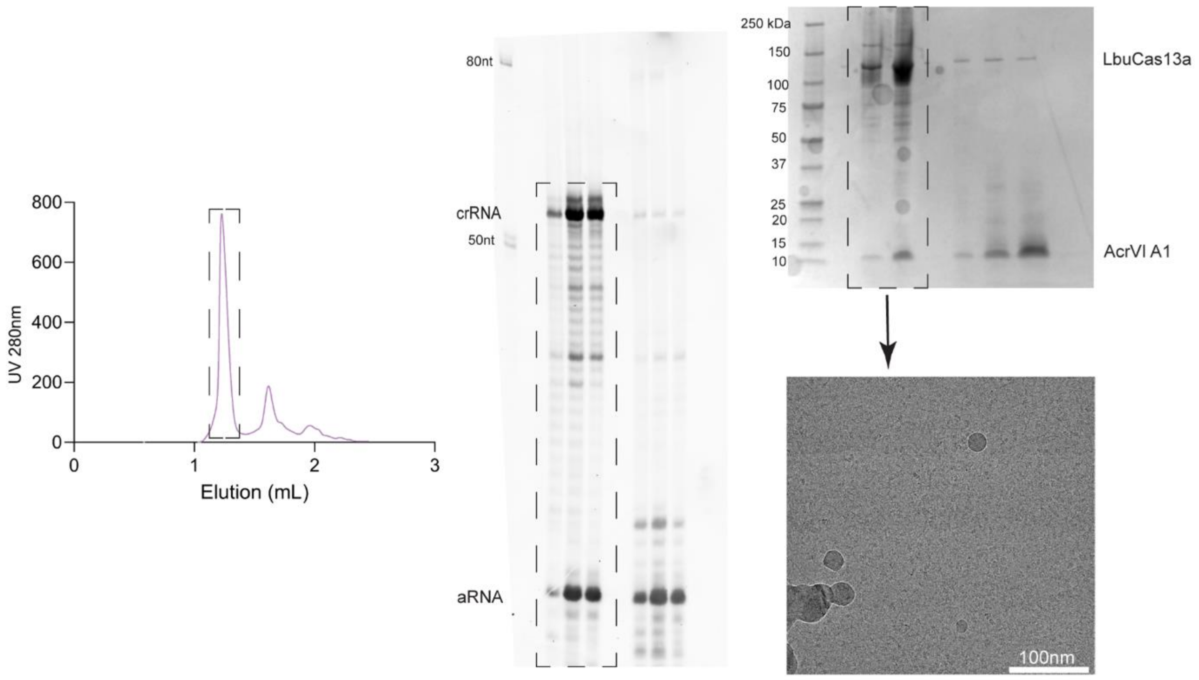

Supplementary Figure 10. LbuCas13a-AIcrVIA1 complex preparation for cryo-EM. The left panel shows the gel filtration profile of the ternary LbuCas13a-AIcrVIA1 complex. Protein, crRNA, and aRNA components are verified by SDS-PAGE (top right) and urea-PAGE (middle right), confirming co-elution of the components for optimal cryo-EM sample preparation (black dashed rectangles). The bottom right panel displays a cryo-EM micrograph.

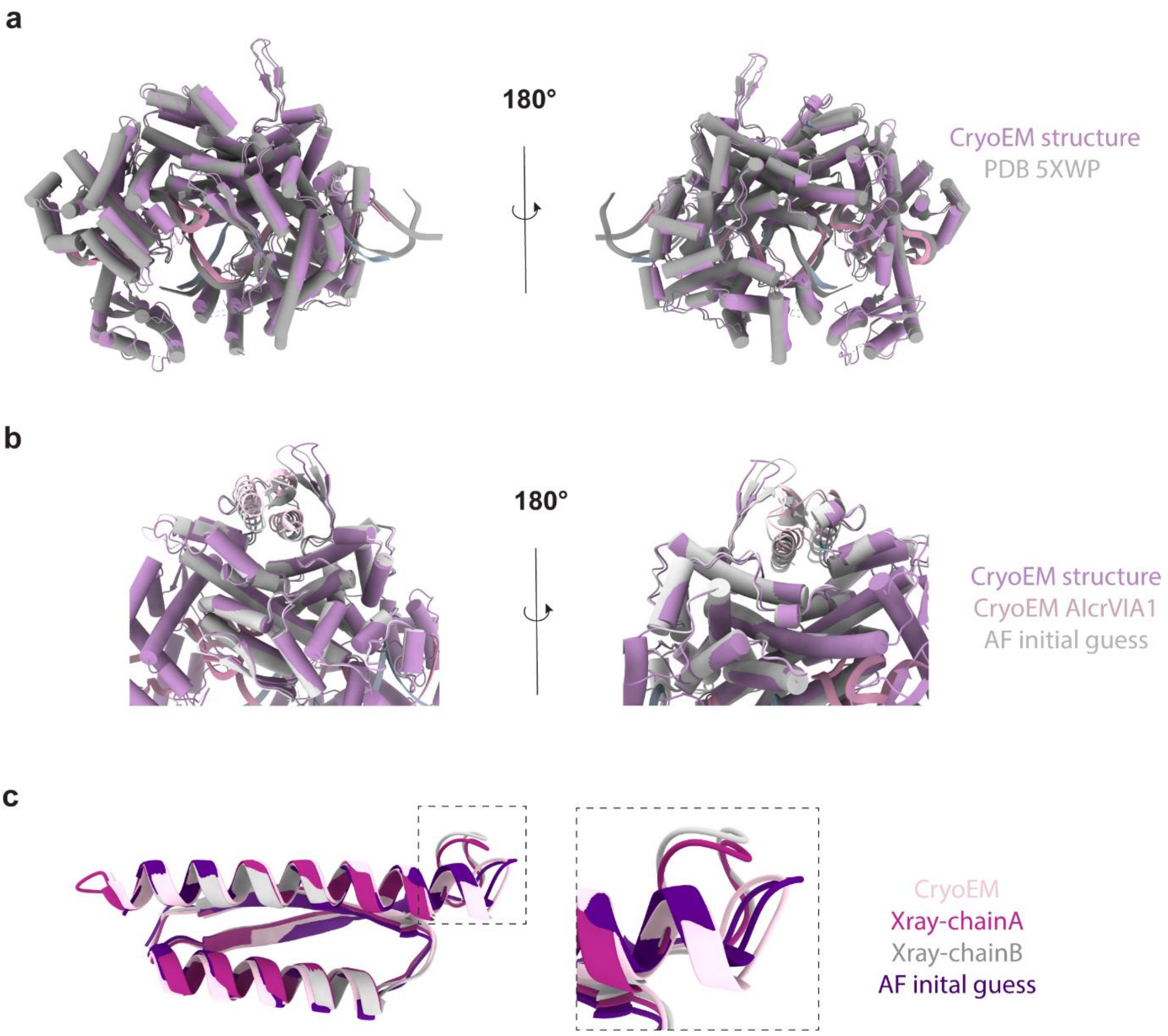

Supplementary Figure 11. Cryo-EM atomic model of the LbuCas13a ternary complex bound to AIcrVIA1 resembles the RF-diffusion prediction. a. Superposition of the LbuCas13a ternary complex atomic model obtained through cryo-EM (cartoon, purple) and the X-ray crystallography structure of the same complex (PDB: 5XWP, cartoon, grey). The structures are highly similar, highlighting the overall structural conservation of the Cas13 ternary complex bound to AIcrVIA1. **b.** Comparison of the cryo-EM model (purple LbuCas13a, pink AIcrVIA1) with the AI-predicted model. The models show striking similarity, with differences in the β-turn region (residues 401-421) and a slight rotation in the AIcrVIA1 position. These deviations can be attributed to the inherent flexibility observed in our heterogeneity studies (**Supplementary Movie 1**). **c.** Cartoon representation of AIcrVIA1 from the cryo-EM atomic model (light pink), X-ray structures (chain A, pink; chain B, grey), and AF initial guess results (violet). The overall alignment between the four structures is consistent, except for a loop region (indicated by black dashed lines), where the cryo-EM structure resembles the AF initial guess more closely than the X-ray structures. This difference may be due to constraints imposed by crystal packing.

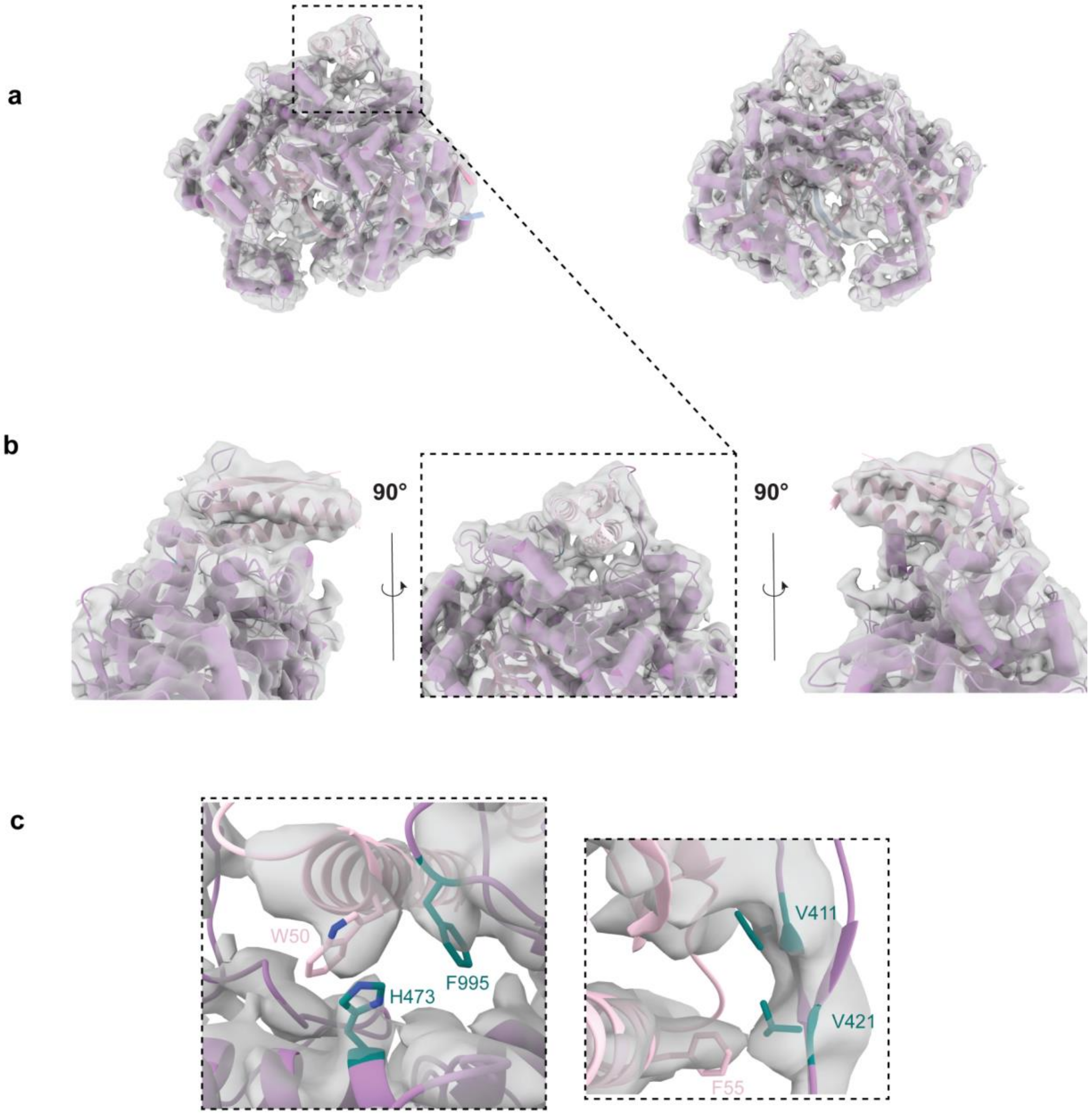

Supplementary Figure 12. Mechanism of AIcrVIA1 inhibition aligns with design predictions. a. Overall fit of the cryo-EM map (transparent surface) for the LbuCas13a-crRNA-aRNA complex bound to AIcrVIA1 (indicated by black dashed lines) atomic model. **b.** Zoomed view of the HEPN1 and HEPN2 domains of LbuCas13a (purple cartoon). AIcrVIA1 is positioned consistently with AI design predictions. **c.** Stick representation of the key interaction hotspots targeted during the AI design workflow (teal). These hotspots are located near aromatic residues, highlighting strong interactions between LbuCas13a and AIcrVIA1. However, structural heterogeneity in this region of the cryo-EM density map (transparent surface) limits high-resolution features.

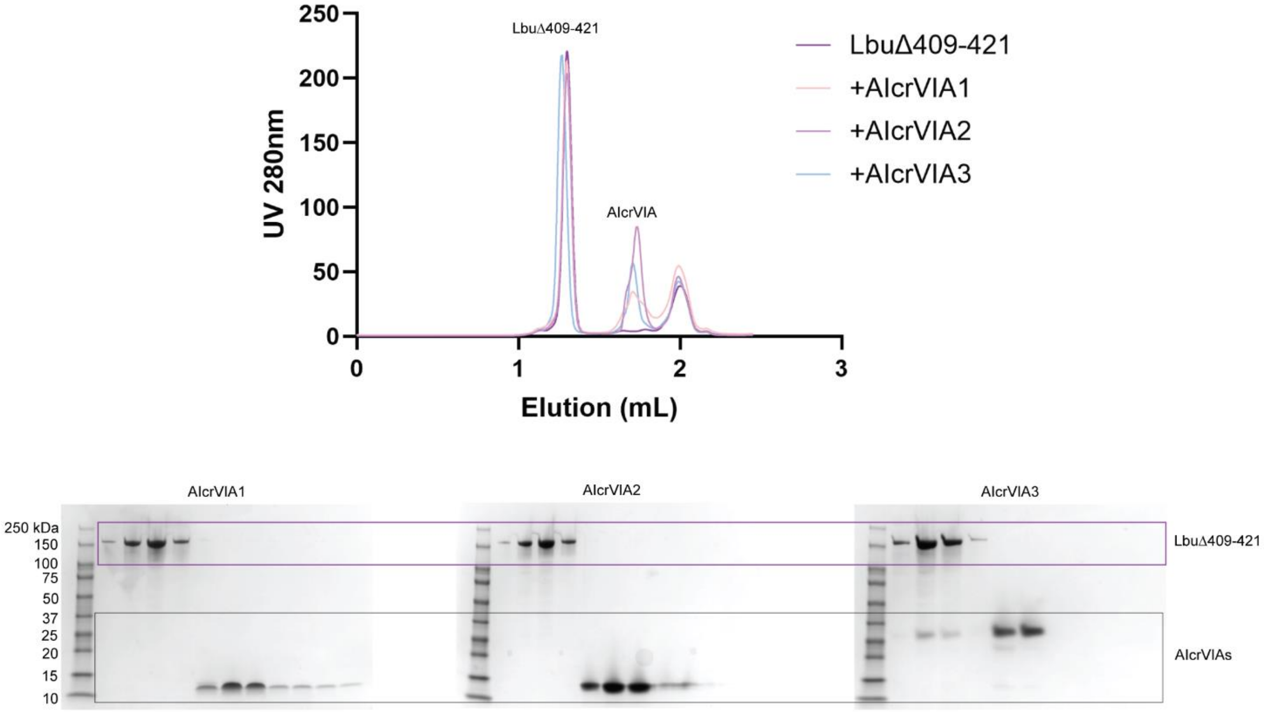

Supplementary Figure 13. LbuCas13aΔ409-421 does not interact with AIcrVIA1 or AIcrVIA2 but retains the ability to interact with AIcrVIA3. The gel filtration chromatogram (top) and corresponding SDS-PAGE analysis (bottom) show protein detection throughout the elution process. The co-elution of LbuCas13aΔ409-421 with AIcrVIA3, but not with AIcrVIA1 or AIcrVIA2, suggests that the β-loop spanning residues 409-421 within the HEPN domain is essential for interaction with AIcrVIA1 and AIcrVIA2. However, AIcrVIA3 retains the capacity to bind the mutant protein.

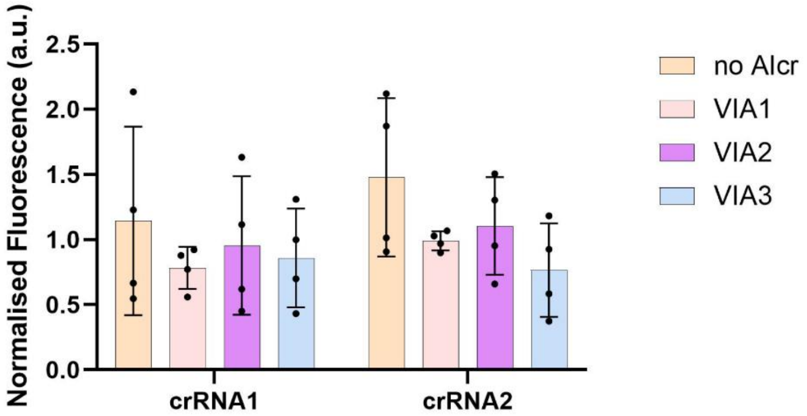

Supplementary Figure 14. Evaluation of potential Cas13a collateral activity in HEK293T cells via mCherry fluorescence. HEK293T cells were co-transfected with LbuCas13a, non-targeting or GFP-targeting crRNAs (crRNA1 and crRNA2), and AIcrVIA constructs (VIA1–3). mCherry was co-expressed to assess possible trans-cleavage (collateral) activity of Cas13a. Fluorescence intensity of mCherry was measured 48 h post-transfection and normalised to the non-targeting crRNA condition. (*n* = 4 with mean ± s.d., biological replicates). Across all conditions, no significant decrease in mCherry fluorescence was observed. These results suggest that collateral cleavage of mCherry, if present, was not reliably detectable under these conditions.

Supplementary Table 1. X-ray Structure Validation and Refinement Statistics Using PHENIX

AlcrVIA1	
**Wavelength**	**0.9536**
**Resolution range**	**42.46 - 1.94 (1.97 - 1.94)**
**Space group**	**P 21 21 21**
**Unit cell**	**33.403 62.9 84.927 90 90 90**
**Total reflections**	**177339 (8398)**
**Unique reflections**	**25959 (1300)**
**Multiplicity**	**6.8 (6.5)**
**Completeness (%)**	**99.37 (90.36)**
**Mean I/sigma(I)**	**10.5 (1.08)**
**Wilson B-factor**	**36.74**
**R-merge**	**0.087 (1.05)**
**CC1/2**	**0.99 (0.66)**
**CC***	**1 (0.89)**
**R-work**	**0.199 (0.288)**
**R-free**	**0.246 (0.343)**
**Number of non-hydrogen atoms**	**1341**
**macromolecules**	**1258**
**ligands**	**1**
**solvent**	**82**
**Protein residues**	**154**
**RMS(bonds)**	**0.003**
**RMS(angles)**	**0.57**
**Ramachandran favored (%)**	**98.67**
**Ramachandran allowed (%)**	**1.33**
**Ramachandran outliers (%)**	**0**
**Rotamer outliers (%)**	**0**
**Clashscore**	**2.8**
**Average B-factor**	**43.25**
**macromolecules**	**43.13**
**ligands**	**69.61**
**solvent**	**44.73**

Supplementary Table 2. Cas13 and AIcrVIA Sequences

**Protein**	**Amino acid sequence**
**6xHis-MBP LbuCas13a**	MKSSHHHHHHGSSMKIEEGKLVIWINGDKGYNGLAEVGKKFEKDTGIKVTVEHPDKLEEKFPQVAATGD<br>GPDIIFWAHDRFGGYAQSGLLAEITPDKAFQDKLYPFTWDAVRYNGKLIAYPIAVEALSLIYNKDLLPNPPK<br>TWEEIPALDKELKAKGKSALMFNLQEPYFTWPLIAADGGYAFKYENGKYDIKDVGVDNAGAKAGLTFLVD<br>LIKNKHMNADTDYSIAEAAFNKGETAMTINGPWAWSNIDTSKVNYGVTVLPTFKGQPSKPFVGVLSAGIN<br>AASPNKELAKEFLENYLLTDEGLEAVNKDKPLGAVALKSYEEELAKDPRIAATMENAQKGEIMPNIPQMS<br>AFWYAVRTAVINAASGRQTVDEALKDAQTNSSSNNNNNNNNNNLGIEENLYFQSNAMKVTKVGGISHKK<br>YTSEGRLVKSESEENRTDERLSALLNMRLDMYIKNPSSTETKENQKRIGKLKKFFSNKMVYLKDNTLSLK<br>NGKKENIDREYSETDILESDVRDKKNFAVLKKIYLNENVNSEELEVFRNDIKKKLNKINSLKYSFEKNKANY<br>QKINENNIEKVEGKSKRNIIYDYYRESAKRDAYVSNVKEAFDKLYKEEDIAKLVLEIENLTKLEKYKIREFYH<br>EIIGRKNDKENFAKIIYEEIQNVNNMKELIEKVPDMSELKKSQVFYKYYLDKEELNDKNIKYAFCHFVEIEMS<br>QLLKNYVYKRLSNISNDKIKRIFEYQNLKKLIENKLLNKLDTYVRNCGKYNYYLQDGEIATSDFIARNRQNE<br>AFLRNIIGVSSVAYFSLRNILETENENDITGRMRGKTVKNNKGEEKYVSGEVDKIYNENKKNEVKENLKMF<br>YSYDFNMDNKNEIEDFFANIDEAISSIRHGIVHFNLELEGKDIFAFKNIAPSEISKKMFQNEINEKKLKLKIFR<br>QLNSANVFRYLEKYKILNYLKRTRFEFVNKNIPFVPSFTKLYSRIDDLKNSLGIYWKTPKTNDDNKTKEIIDA<br>QIYLLKNIYYGEFLNYFMSNNGNFFEISKEIIELNKNDKRNLKTGFYKLQKFEDIQEKIPKEYLANIQSLYMIN<br>AGNQDEEEKDTYIDFIQKIFLKGFMTYLANNGRLSLIYIGSDEETNTSLAEKKQEFDKFLKKYEQNNNIKIP<br>YEINEFLREIKLGNILKYTERLNMFYLILKLLNHKELTNLKGSLEKYQSANKEEAFSDQLELINLLNLDNNRV<br>TEDFELEADEIGKFLDFNGNKVKDNKELKKFDTNKIYFDGENIIKHRAFYNIKKYGMLNLLEKIADKAGYKIS<br>IEELKKYSNKKNEIEKNHKMQENLHRKYARPRKDEKFTDEDYESYKQAIENIEEYTHLKNKVEFNELNLLQ<br>GLLLRILHRLVGYTSIWERDLRFRLKGEFPENQYIEEIFNFENKKNVKYKGGQIVEKYIKFYKELHQNDEVK<br>INKYSSANIKVLKQEKKDLYIRNYIAHFNYIPHAEISLLEVLENLRKLLSYDRKLKNAVMKSVVDILKEYGFV<br>ATFKIGADKKIGIQTLESEKIVHLKNLKKKKLMTDRNSEELCKLVKIMFEYKMEEKKSEN
**6xHis-MBP LbuCas13a Δ409-421**	MKSSHHHHHHGSSMKIEEGKLVIWINGDKGYNGLAEVGKKFEKDTGIKVTVEHPDKLEEKFPQVAATG<br>DGPDIIFWAHDRFGGYAQSGLLAEITPDKAFQDKLYPFTWDAVRYNGKLIAYPIAVEALSLIYNKDLLPN<br>PPKTWEEIPALDKELKAKGKSALMFNLQEPYFTWPLIAADGGYAFKYENGKYDIKDVGVDNAGAKAGL<br>TFLVDLIKNKHMNADTDYSIAEAAFNKGETAMTINGPWAWSNIDTSKVNYGVTVLPTFKGQPSKPFVG<br>VLSAGINAASPNKELAKEFLENYLLTDEGLEAVNKDKPLGAVALKSYEEELAKDPRIAATMENAQKGEI<br>MPNIPQMSAFWYAVRTAVINAASGRQTVDEALKDAQTNSSSNNNNNNNNNNLGIEENLYFQSNAMKV<br>TKVGGISHKKYTSEGRLVKSESEENRTDERLSALLNMRLDMYIKNPSSTETKENQKRIGKLKKFFSNK<br>MVYLKDNTLSLKNGKKENIDREYSETDILESDVRDKKNFAVLKKIYLNENVNSEELEVFRNDIKKKLNKI<br>NSLKYSFEKNKANYQKINENNIEKVEGKSKRNIIYDYYRESAKRDAYVSNVKEAFDKLYKEEDIAKLVLEI<br>ENLTKLEKYKIREFYHEIIGRKNDKENFAKIIYEEIQNVNNMKELIEKVPDMSELKKSQVFYKYYLDKEEL<br>NDKNIKYAFCHFVEIEMSQLLKNYVYKRLSNISNDKIKRIFEYQNLKKLIENKLLNKLDTYVRNCGKYNYY<br>LQDGEIATSDFIARNRQNEAFLRNIIGVSSVAYFSLRNILETENENDITGRMRGSGEVDKIYNENKKNEV<br>KENLKMFYSYDFNMDNKNEIEDFFANIDEAISSIRHGIVHFNLELEGKDIFAFKNIAPSEISKKMFQNEINE<br>KKLKLKIFRQLNSANVFRYLEKYKILNYLKRTRFEFVNKNIPFVPSFTKLYSRIDDLKNSLGIYWKTPKTN<br>DDNKTKEIIDAQIYLLKNIYYGEFLNYFMSNNGNFFEISKEIIELNKNDKRNLKTGFYKLQKFEDIQEKIPK<br>EYLANIQSLYMINAGNQDEEEKDTYIDFIQKIFLKGFMTYLANNGRLSLIYIGSDEETNTSLAEKKQEFDK<br>FLKKYEQNNNIKIPYEINEFLREIKLGNILKYTERLNMFYLILKLLNHKELTNLKGSLEKYQSANKEEAFSD<br>QLELINLLNLDNNRVTEDFELEADEIGKFLDFNGNKVKDNKELKKFDTNKIYFDGENIIKHRAFYNIKKYG<br>MLNLLEKIADKAGYKISIEELKKYSNKKNEIEKNHKMQENLHRKYARPRKDEKFTDEDYESYKQAIENIE<br>EYTHLKNKVEFNELNLLQGLLLRILHRLVGYTSIWERDLRFRLKGEFPENQYIEEIFNFENKKNVKYKGG<br>QIVEKYIKFYKELHQNDEVKINKYSSANIKVLKQEKKDLYIRNYIAHFNYIPHAEISLLEVLENLRKLLSYD<br>RKLKNAVMKSVVDILKEYGFVATFKIGADKKIGIQTLESEKIVHLKNLKKKKLMTDRNSEELCKLVKIMFE<br>YKMEEKKSEN
**6xHis-MBP LbaCas13a**	MKSSHHHHHHGSSMKIEEGKLVIWINGDKGYNGLAEVGKKFEKDTGIKVTVEHPDKLEEKFPQVAATG<br>DGPDIIFWAHDRFGGYAQSGLLAEITPDKAFQDKLYPFTWDAVRYNGKLIAYPIAVEALSLIYNKDLLPN<br>PPKTWEEIPALDKELKAKGKSALMFNLQEPYFTWPLIAADGGYAFKYENGKYDIKDVGVDNAGAKAGL<br>TFLVDLIKNKHMNADTDYSIAEAAFNKGETAMTINGPWAWSNIDTSKVNYGVTVLPTFKGQPSKPFVG<br>VLSAGINAASPNKELAKEFLENYLLTDEGLEAVNKDKPLGAVALKSYEEELAKDPRIAATMENAQKGEI<br>MPNIPQMSAFWYAVRTAVINAASGRQTVDEALKDAQTNSSSNNNNNNNNNNLGIEENLYFQSNAMKI<br>SKVREENRGAKLTVNAKTAVVSENRSQEGILYNDPSRYGKSRKNDEDRDRYIESRLKSSGKLYRIFNE<br>DKNKRETDELQWFLSEIVKKINRRNGLVLSDMLSVDDRAFEKAFEKYAELSYTNRRNKVSGSPAFETC<br>GVDAATAERLKGIISETNFINRIKNNIDNKVSEDIIDRIIAKYLKKSLCRERVKRGLKKLLMNAFDLPYSDP<br>DIDVQRDFIDYVLEDFYHVRAKSQVSRSIKNMNMPVQPEGDGKFAITVSKGGTESGNKRSAEKEAFKK<br>FLSDYASLDERVRDDMLRRMRRLVVLYFYGSDDSKLSDVNEKFDVWEDHAARRVDNREFIKLPLENK<br>LANGKTDKDAERIRKNTVKELYRNQNIGCYRQAVKAVEEDNNGRYFDDKMLNMFFIHRIEYGVEKIYA<br>NLKQVTEFKARTGYLSEKIWKDLINYISIKYIAMGKAVYNYAMDELNASDKKEIELGKISEEYLSGISSFD<br>YELIKAEEMLQRETAVYVAFAARHLSSQTVELDSENSDFLLLKPKGTMDKNDKNKLASNNILNFLKDKE<br>TLRDTILQYFGGHSLWTDFPFDKYLAGGKDDVDFLTDLKDVIYSMRNDSFHYATENHNNGKWNKELIS<br>AMFEHETERMTVVMKDKFYSNNLPMFYKNDDLKKLLIDLYKDNVERASQVPSFNKVFVRKNFPALVR<br>DKDNLGIELDLKADADKGENELKFYNALYYMFKEIYYNAFLNDKNVRERFITKATKVADNYDRNKERNL<br>KDRIKSAGSDEKKKLREQLQNYIAENDFGQRIKNIVQVNPDYTLAQICQLIMTEYNQQNNGCMQKKSA<br>ARKDINKDSYQHYKMLLLVNLRKAFLEFIKENYAFVLKPYKHDLCDKADFVPDFAKYVKPYAGLISRVA<br>GSSELQKWYIVSRFLSPAQANHMLGFLHSYKQYVWDIYRRASETGTEINHSIAEDKIAGVDITDVDAVID<br>LSVKLCGTISSEISDYFKDDEVYAEYISSYLDFEYDGGNYKDSLNRFCNSDAVNDQKVALYYDGEHPKL

	NRNIILSKLYGERRFLEKITDRVSRSDIVEYYKLKKETSQYQTKGIFDSEDEQKNIKKFQEMKNIVEFRDL<br>MDYSEIADELQGQLINWIYLRERDLMNFQLGYHYACLNNDSNKQATYVTLDYQGKKNRKINGAILYQIC<br>AMYINGLPLYYVDKDSSEWTVSDGKESTGAKIGEFYRYAKSFENTSDCYASGLEIFENISEHDNITELR<br>NYIEHFRYYSSFDRSFLGIYSEVFDRFFTYDLKYRKNVPTILYNILLQHFVNVRFEFVSGKKMIGIDKKDR<br>KIAKEKECARITIREKNGVYSEQFTYKLKNGTVYVDARDKRYLQSIIRLLFYPEKVNMDEMIEVKEKKKP<br>SDNNTGKGYSKRDRQQDRKEYDKYKEKKKKEGNFLSGMGGNINWDEINAQLKN
**6xHis-SUMO TccCas13a**	MGSSHHHHHHSSGLVPRGSHMSGSAAGGEEDKKPAGGEGGGAHINLKVKGQDGNEVFFRIKRSTQ<br>LKKLMNAYCDRQSVDMTAIAFLFDGRRLRAEQTPDELEMEDGDEIDAMLHQTGGMKITKRKWGEHHP<br>PLYFYRDEDSGRLLAQNDRKQDYTDTLFNDIAQDTFERSLRNRLLKTPEKGDKRFYSNEIVKLVEKLC<br>QGADVAEIMKSMERNEKLRPKNEKEIKNLKKQLDGTLSEYGKRYTAPEGAMTLNDALFYLVEGNPLKQ<br>AMAKAELGKIREALIKEKENRINRVRYSIKNNKIPLRIQEDGGITPNNDRAAWLLGLMKPADPAKGITDC<br>YPLLGELEEVFDFDKLSKTLHEKISRCQGRPRSIAMAVDEALKQYLRELWEKSPSRQQDLKYYFQAVQ<br>EYFKDNFPIRTKRMGARLRQELLKDKTSLSRLLEPKHMANAVRRRLINQSTQMHILYGKLYAYCCGED<br>GRLLVNSETLQRIQVHEAVKKQAMTAVLWSISRLRYFYQFEDGDILSNKNPIKDFRDKFLRDTNKYTHE<br>DVEACKEKLQDFFPLKELQEKIKEDAKGLQETDNKQADTTDFKAIGHIVRDDRKLCNQLLAECVSCIGE<br>LRHHIFHYKNVTLIQALKRIADKVKPEDLSVLRAIYLLDRRNLKKAFAKRISSMNLPLYYREDLLSRIFKKE<br>GTAFFLYSAKIQMTPSFQRVYERGKNLRREFECERMKAEASNGQNGQDGDRLKWFRQLAAGDSADT<br>HFNWAVEAYAESAADVENNVEFDTDVDAQRALRNLLLLIYRHHFLPEVQKDETLVTGKIHKVLERNRQ<br>LSEGQGPNQGKAHGYSVIEELYHEGMPLSDLMKQLQRRISETERESRELAQEKTDYAQRFILDIFAEA<br>FNDFLEAHYGEEYLEIMSPRKDAEAAKKWVKESKTVDLKTSIDEKEPEGHLLVLYPVLRLLDERELGEL<br>QQQMIRYRTSLASWQGESNFSEEIRIAGQIEELTELVKLTEPEPQFAEEVWGKRAKEAFEDFIEGNMK<br>NYEAFYLQSDNNTPVYRRNMSRLLRSGLMGVYQKVLASHKQALKRDYLLWSEKHWNVKDENGADIS<br>SAEQAQCLLQRLHRKYAESPSRFTEEDCKLYEKVLRRLEDYNQAVKNLSFSSLYEICVLNLEILSRWVG<br>FVQDWERDMYFLLLAWVRQGKLDGIKEEDVRDIFSEGNIIRNLVDTLKGENMNAFESVYFPENKGSKY<br>LGVRNDVAHLDLMRKNGWRLEAGKTCSVMEDYINRLRFLLSYDQKRMNAVTKTLQQIFDRHKVKIRFT<br>VEKGGMLKIEDVTADKIVHLKGSRLSGIEIPSHGERFIDTLKALMVYPRG
**RfxCas13d-6xHis**	MIEKKKSFAKGMGVKSTLVSGSKVYMTTFAEGSDARLEKIVEGDSIRSVNEGEAFSAEMADKNAGYKI<br>GNAKFSHPKGYAVVANNPLYTGPVQQDMLGLKETLEKRYFGESADGNDNICIQVIHNILDIEKILAEYIT<br>NAAYAVNNISGLDKDIIGFGKFSTVYTYDEFKDPEHHRAAFNNNDKLINAIKAQYDEFDNFLDNPRLGYF<br>GQAFFSKEGRNYIINYGNECYDILALLSGLRHWVVHNNEEESRISRTWLYNLDKNLDNEYISTLNYLYD<br>RITNELTNSFSKNSAANVNYIAETLGINPAEFAEQYFRFSIMKEQKNLGFNITKLREVMLDRKDMSEIRK<br>NHKVFDSIRTKVYTMMDFVIYRYYIEEDAKVAAANKSLPDNEKSLSEKDIFVINLRGSFNDDQKDALYYD<br>EANRIWRKLENIMHNIKEFRGNKTREYKKKDAPRLPRILPAGRDVSAFSKLMYALTMFLDGKEINDLLTT<br>LINKFDNIQSFLKVMPLIGVNAKFVEEYAFFKDSAKIADELRLIKSFARMGEPIADARRAMYIDAIRILGTN<br>LSYDELKALADTFSLDENGNKLKKGKHGMRNFIINNVISNKRFHYLIRYGDPAHLHEIAKNEAVVKFVLG<br>RIADIQKKQGQNGKNQIDRYYETCIGKDKGKSVSEKVDALTKIITGMNYDQFDKKRSVIEDTGRENAER<br>EKFKKIISLYLTVIYHILKNIVNINARYVIGFHCVERDAQLYKEKGYDINLKKLEEKGFSSVTKLCAGIDETA<br>PDKRKDVEKEMAERAKESIDSLESANPKLYANYIKYSDEKKAEEFTRQINREKAKTALNAYLRNTKWN<br>VIIREDLLRIDNKTCTLFRNKAVHLEVARYVHAYINDIAEVNSYFQLYHYIMQRIIMNERYEKSSGKVSEY<br>FDAVNDEKKYNDRLLKLLCVPFGYCIPRFKNLSIEALFDRNEAAKFDKEKKKVSGNSSRGSHHHHHH
**AIcrVIA1-6xHis**	MKTIKVDVIVVGDDEELVEEYKKEAELIGKEYGVKIEVEPYFLEEGKFPWLDVDFAYNTTQEELDKAEK<br>EAKKIAGSHHHHHH
**AIcrVIA2-6xHis**	MNEKKEKARKEMKEIAEKAIKEIKKDPEKALEIAVKAVEEIGEIANESGDYKSGEENAWKVAEEYAKVT<br>GDDDTAYDLMLAGDMIIDGKDAEEGYEILKELGSHHHHHH
**AIcrVIA3-6xHIs**	MAPKKYVVTVTIPVTDADLTVFLVVDIIVYAEKLGGTVTITAVKSENDSYSVTLEDLDKAAEELEKVGGSI<br>VLTVTFDNKEKAEKVAEFAYLKAEEYNLKVDVEVKEELGSHHHHHH

Source Data files for Supplementary Figures

Supplementary Figure 10

Source Data Supplementary Figure 10

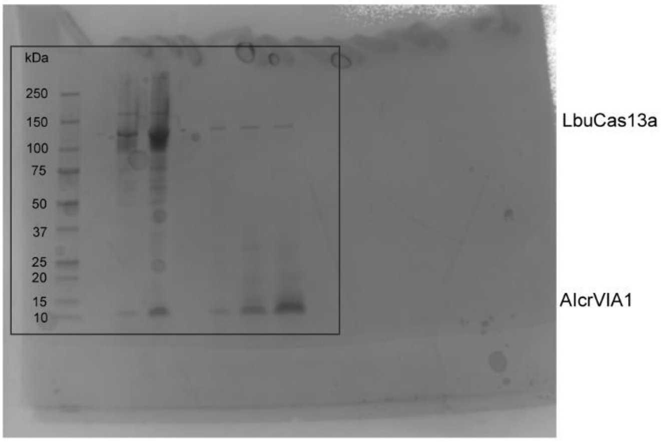

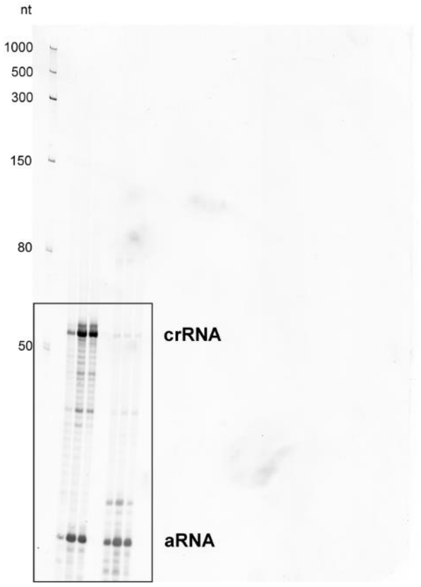

Supplementary Figure 13

Source Data Supplementary Figure 13

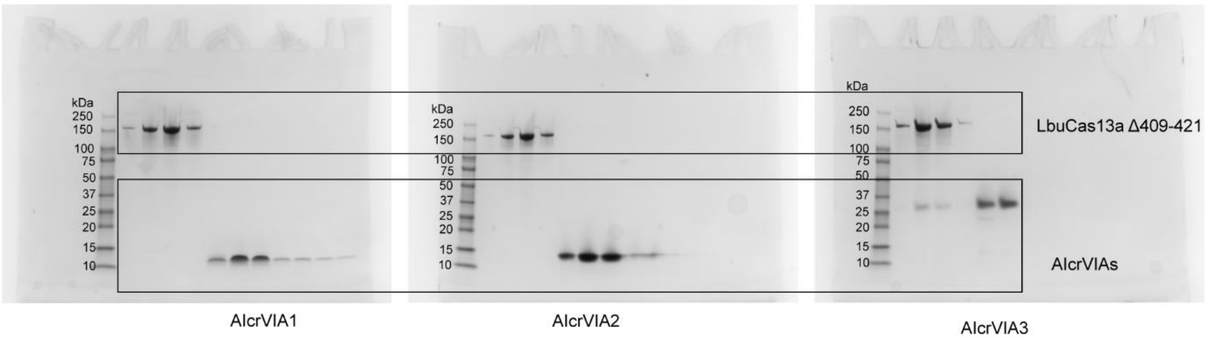

Supplement: Supplementary file 1 — Supplementary Figs. 1–14, Tables 1 and 2 and Source data for Supplementary Figs. 10 and 13. [file 41589_2025_2136_MOESM1_ESM.pdf]
