# Supplementary material for: De novo design of potent CRISPR–Cas13 inhibitors
Source: Nat Chem Biol. 2026 Jan 26;22(8):1342–50. doi: 10.1038/s41589-025-02136-3 (PMC13423800; doi:10.1038/s41589-025-02136-3)
Supplement: Supplementary file 2 — Reporting Summary [file 41589_2025_2136_MOESM2_ESM.pdf]

nature portfolio

Corresponding author(s): A/Prof. Gavin Knott
Dr. Rhys Grinter
Dr. Cyntia Taveneau

Last updated by author(s): 9/11/2025

Reporting Summary

Nature Portfolio wishes to improve the reproducibility of the work that we publish. This form provides structure for consistency and transparency in reporting. For further information on Nature Portfolio policies, see our Editorial Policies and the Editorial Policy Checklist.

Statistics

For all statistical analyses, confirm that the following items are present in the figure legend, table legend, main text, or Methods section.

n/a	Confirmed	
☐	☒	The exact sample size (*n*) for each experimental group/condition, given as a discrete number and unit of measurement
☐	☒	A statement on whether measurements were taken from distinct samples or whether the same sample was measured repeatedly
☐	☒	The statistical test(s) used AND whether they are one- or two-sided<br>*Only common tests should be described solely by name; describe more complex techniques in the Methods section.*
☒	☐	A description of all covariates tested
☐	☒	A description of any assumptions or corrections, such as tests of normality and adjustment for multiple comparisons
☐	☒	A full description of the statistical parameters including central tendency (e.g. means) or other basic estimates (e.g. regression coefficient) AND variation (e.g. standard deviation) or associated estimates of uncertainty (e.g. confidence intervals)
☒	☐	For null hypothesis testing, the test statistic (e.g. F, t, r) with confidence intervals, effect sizes, degrees of freedom and P value noted<br>*Give P values as exact values whenever suitable.*
☒	☐	For Bayesian analysis, information on the choice of priors and Markov chain Monte Carlo settings
☒	☐	For hierarchical and complex designs, identification of the appropriate level for tests and full reporting of outcomes
☒	☐	Estimates of effect sizes (e.g. Cohen's *d*, Pearson's *r*), indicating how they were calculated

Our web collection on statistics for biologists contains articles on many of the points above.

Software and code

Policy information about availability of computer code

Data collection	CryoEM data Collection : EPU
Data analysis	AIcrVIs AI-design : open source software (RF-Diffusion1.1 and protein MPNN) https://github.com/RosettaCommons/RFdiffusion<br>Binder Tree : FoldSeek Search Server - Open source, used online (https://search.foldseek.com/)<br>Circular Dichroism : JASCO spectra manager (v2)<br>Protein Prediction : AlphaFold 2 (open source, https://github.com/google-deepmind/alphafold)<br>CryoEM structure : MotionCor2 1.4.5, CryoSPARC 4.5.2, Topaz 0.2.5, Coot v0.9.4, Phenix v1.18.2<br>X-Ray Crystallography : Phenix 1.20.1, WinCoot 0.9.8.93, XDS<br>Structure visualization : ChimeraX<br>Data analysis (graphs) : GraphPad PRISM (v10.4, v9)<br>FACS : FlowJo V10

For manuscripts utilizing custom algorithms or software that are central to the research but not yet described in published literature, software must be made available to editors and reviewers. We strongly encourage code deposition in a community repository (e.g. GitHub). See the Nature Portfolio guidelines for submitting code & software for further information.

Data

Policy information about availability of data

All manuscripts must include a data availability statement. This statement should provide the following information, where applicable:

- Accession codes, unique identifiers, or web links for publicly available datasets
- A description of any restrictions on data availability
- For clinical datasets or third party data, please ensure that the statement adheres to our policy

The cryo-EM and X-ray crystallography structures have been deposited in the Protein Data Bank under accession codes 9MVS (cryo-EM) and 9MVR (X-ray). Plasmids encoding AlcrVIA1–VIA3 are available from Addgene (pBAD vectors carrying the binder proteins: IDs 231127, 231126, and 231125; pET29b+ vectors: IDs 234054, 234053, and 234052). Source data include the raw results from activity assays across 96 designed proteins (cell-free and semi-purified), as well as uncropped gels and western blots, and are provided with this paper as Source Data files.

Research involving human participants, their data, or biological material

Policy information about studies with human participants or human data. See also policy information about sex, gender (identity/presentation), and sexual orientation and race, ethnicity and racism.

Reporting on sex and gender	n/a
Reporting on race, ethnicity, or other socially relevant groupings	n/a
Population characteristics	n/a
Recruitment	n/a
Ethics oversight	n/a

Note that full information on the approval of the study protocol must also be provided in the manuscript.

Field-specific reporting

Please select the one below that is the best fit for your research. If you are not sure, read the appropriate sections before making your selection.

☒ Life sciences ☐ Behavioural & social sciences ☐ Ecological, evolutionary & environmental sciences

For a reference copy of the document with all sections, see nature.com/documents/nr-reporting-summary-flat.pdf

Life sciences study design

All studies must disclose on these points even when the disclosure is negative.

Sample size	The sample sizes were determined to match the standards in comparable studies available in the literature (Chunlong Xu et at, Nat Methods, 2021; Hu et al, Nat. Struct. Mol. Biol).
Data exclusions	Experiments and protocols were optimized in pilot assays before generating high-quality publication data. No data were excluded from the analysis.
Replication	All experiments were repeated at least 3 times as biological or technical replicates as indicated in the manuscript. All experiments were reproducible.
Randomization	No randomization was used in this study. Due to the small sample, randomization was not relevant for this study. Covariates were controlled for by running controls in parallel whenever is applicable. Appropriate controls (e.g. non targeting crRNAs, loading controls in WB) were used throughout the study.
Blinding	No blinding was used in this study.

Reporting for specific materials, systems and methods

We require information from authors about some types of materials, experimental systems and methods used in many studies. Here, indicate whether each material, system or method listed is relevant to your study. If you are not sure if a list item applies to your research, read the appropriate section before selecting a response.

Materials & experimental systems

n/a	Involved in the study
[ ]	[x] Antibodies
[ ]	[x] Eukaryotic cell lines
[x]	[ ] Palaeontology and archaeology
[x]	[ ] Animals and other organisms
[x]	[ ] Clinical data
[x]	[ ] Dual use research of concern
[x]	[ ] Plants

Methods

n/a	Involved in the study
[x]	[ ] ChIP-seq
[ ]	[x] Flow cytometry
[x]	[ ] MRI-based neuroimaging

Antibodies

Antibodies used	mouse anti-HA mAb (Cell Signaling, #2367) 1:2000 dilution ; mouse anti-β-Actin mAb (Sigma-Aldrich, A2228) 1:10000 dilution ; rabbit anti-mouse Immunoglobulins/HRP (Dako, P0260) 1:10000 dilution
Validation	The antibodies are validated by the manufacturers and by our lab in optimization assays.

Eukaryotic cell lines

Policy information about cell lines and Sex and Gender in Research

Cell line source(s)	HEK293T cell line (ATCC CRL-3216)
Authentication	Cell lines were authenticated by the supplier ATCC. We did not perform any additional authentication upon reception. We made a bulk stock of this cell line after recovering from the original frozen vials. We discard the cells after ~20 passages, and thaw new cells from the liquid nitrogen stocks. Cell morphology was monitored at each passage by microscopy.
Mycoplasma contamination	HEK293T cells are regularly tested for mycoplasma, and only negative samples are cultured.
Commonly misidentified lines (See ICLAC register)	N/A

Plants

Seed stocks	n/a
Novel plant genotypes	n/a
Authentication	n/a

Flow Cytometry

Plots

Confirm that:

[x] The axis labels state the marker and fluorochrome used (e.g. CD4-FITC).

[x] The axis scales are clearly visible. Include numbers along axes only for bottom left plot of group (a 'group' is an analysis of identical markers).

[x] All plots are contour plots with outliers or pseudocolor plots.

[x] A numerical value for number of cells or percentage (with statistics) is provided.

Methodology

Sample preparation	HEK293T cells (ATCC CRL-3216) were resuspended in 100 µL of PBS containing 2% FBS (v/v) for flow cytometry analysis.
Instrument	FACS Symphony Cell Analyzer A5

Software	FlowJo V10
Cell population abundance	A total of approximately 17,000 cells were collected and analyzed. Of these, around 60% were gated as morphologically viable, and within this population, approximately 80% were identified as singlets. Only these singlet, viable cells were analyzed for their GFP fluorescence intensity using the 488 nm laser channel (515/20 filter).
Gating strategy	Cell morphology was first gated based on size and granularity using SSC-A and FSC-A parameters. Singlets were then identified by gating on FSC-H versus FSC-A to exclude doublets. The gating threshold for GFP-positive fluorescence was determined using negative control samples lacking GFP expression.

☒ Tick this box to confirm that a figure exemplifying the gating strategy is provided in the Supplementary Information.
