# Supplementary figures and images for: De novo design of potent CRISPR–Cas13 inhibitors
Source: Nat Chem Biol. 2026 Jan 26;22(8):1342–50. doi: 10.1038/s41589-025-02136-3 (PMC13423800; doi:10.1038/s41589-025-02136-3)

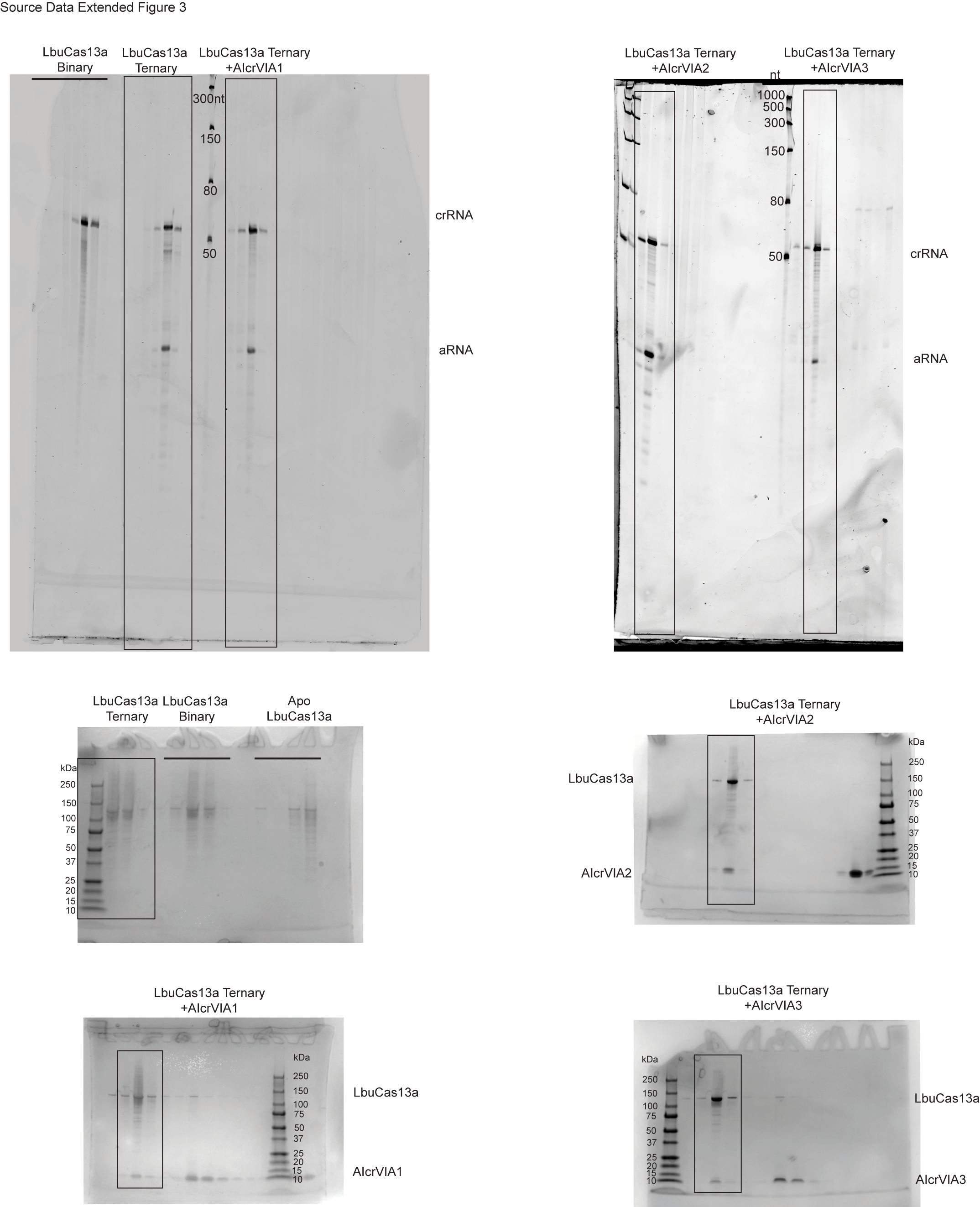

Supplement: Supplementary file 6 — Unprocessed and uncropped urea gels and SDS–PAGE. [file 41589_2025_2136_MOESM6_ESM.tif]

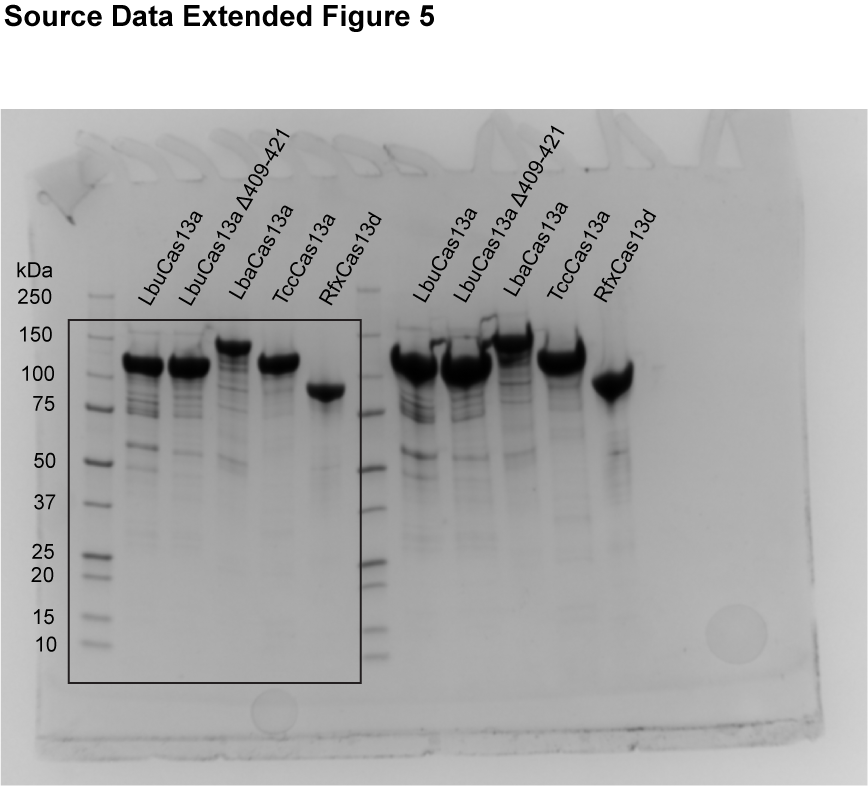

Supplement: Supplementary file 7 — Unprocessed and uncropped SDS–PAGE. [file 41589_2025_2136_MOESM7_ESM.tif]

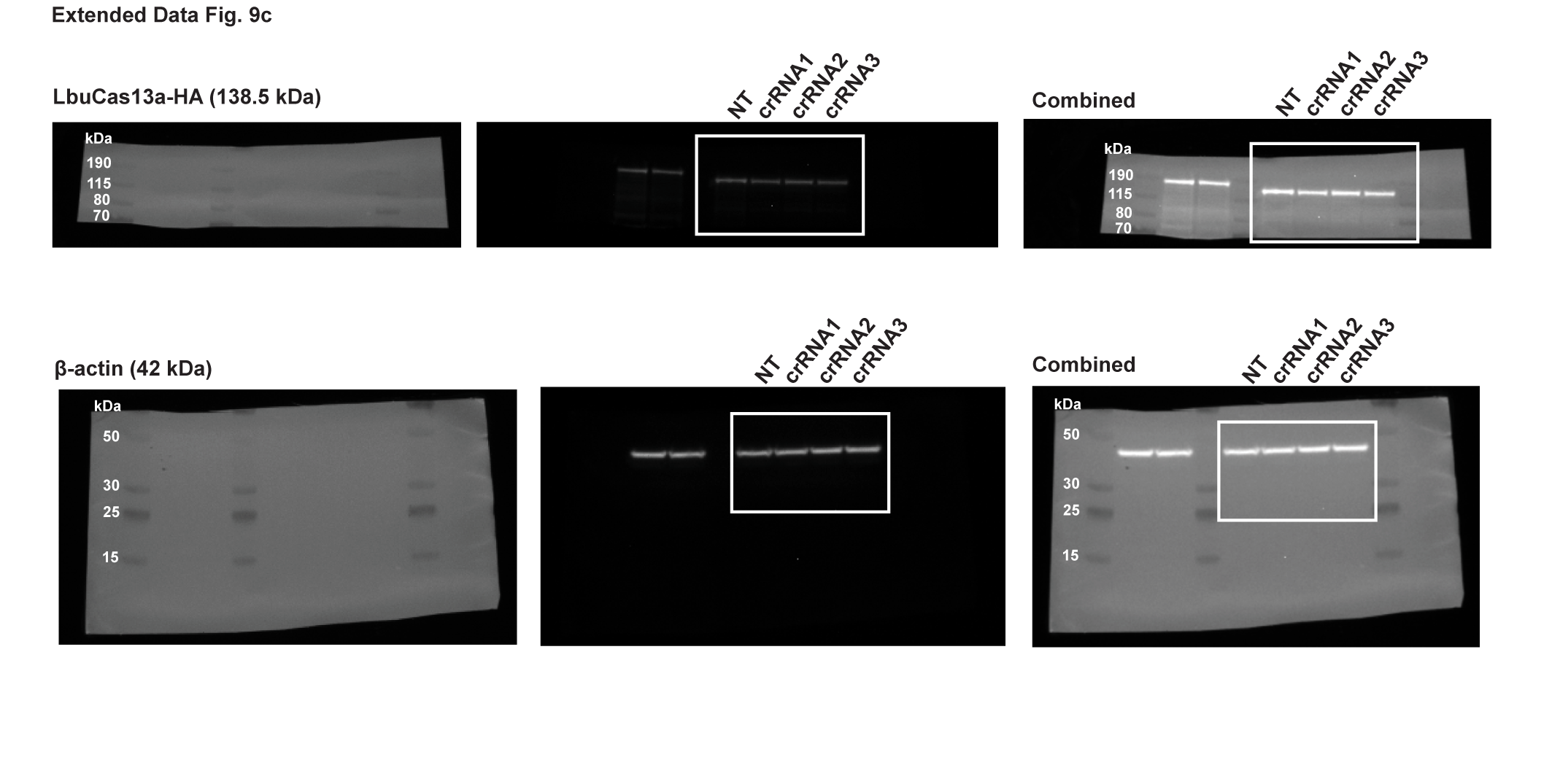

Supplement: Supplementary file 8 — Unprocessed and uncropped western blots. [file 41589_2025_2136_MOESM8_ESM.tif]
